# Insect species richness affects plant responses to multi‐herbivore attack

**DOI:** 10.1111/nph.17228

**Published:** 2021-02-15

**Authors:** Maite Fernández de Bobadilla, Mitchel E. Bourne, Janneke Bloem, Sarah N. Kalisvaart, Gerrit Gort, Marcel Dicke, Erik H. Poelman

**Affiliations:** ^1^ Laboratory of Entomology Wageningen University and Research Centre Droevendaalsesteeg 1 Wageningen 6708PB the Netherlands; ^2^ Biometris, Wageningen University and Research Centre Droevendaalsesteeg 1 Wageningen 6708PB the Netherlands

**Keywords:** herbivore species richness, herbivore‐induced plant gene expression, multi‐herbivore attack, phytohotmonal crosstalk, plant‐mediated interactions, *Plutella xylostella*

## Abstract

- Plants are often attacked by multiple insect herbivores. How plants deal with an increasing richness of attackers from a single or multiple feeding guilds is poorly understood.
- We subjected black mustard (*Brassica nigra*) plants to 51 treatments representing attack by an increasing species richness (one, two or four species) of either phloem feeders, leaf chewers, or a mix of both feeding guilds when keeping total density of attackers constant and studied how this affects plant resistance to subsequent attack by caterpillars of the diamondback moth (*Plutella xylostella*).
- Increased richness in phloem‐feeding attackers compromised resistance to *P. xylostella*. By contrast, leaf chewers induced a stronger resistance to subsequent attack by caterpillars of *P. xylostella* while species richness did not play a significant role for chewing herbivore induced responses. Attack by a mix of herbivores from different feeding guilds resulted in plant resistance similar to resistance levels of plants that were not previously exposed to herbivory.
- We conclude that *B. nigra* plants channel their defence responses stronger towards a feeding‐guild specific response when under multi‐species attack by herbivores of the same feeding guild, but integrate responses when simultaneously confronted with a mix of herbivores from different feeding guilds.

## Abstract 2

See also the Commentary on this article by Croy *et al*., **231**: 2110–2112.

## Introduction

Plants interact with a community of organisms, from which insects are among the most prominent members. Some of these interactions benefit plant fitness, such as interactions with pollinators or plant growth‐promoting microorganisms (Berg, 2009; Pineda *et al*., 2010; Giron *et al*., 2018). However, many plant interactions negatively impact plant fitness because they involve organisms that consume plant tissues, such as insect herbivores. In natural ecosystems, plants are commonly attacked by multiple insect herbivores and are under selection to optimize their defences against their community of attackers to maximize their fitness (Lankau & Strauss, 2008; Wise & Rausher, 2013; Poelman & Kessler, 2016). Because the production and maintenance of plant defences is energetically costly, most defence mechanisms are inducible (i.e. only activated upon herbivore feeding) to save the costs of defence in the absence of herbivores (Karban, 2019). To deal with a suite of attackers that may all require different defensive traits, plants have evolved mechanisms to recognize the specific attacker by its feeding guild (e.g. leaf chewer or phloem feeder), feeding pattern, feeding position, or elicitors in its saliva, and tailor the induced defence phenotype to the specific attacking herbivore (Acevedo *et al*., 2015; Züst & Agrawal, 2016). In this context, plants use a network of phytohormones to regulate responses to specific herbivores with jasmonic acid (JA) signalling being dominantly involved in resistance to leaf chewing herbivores and salicylic acid (SA) to phloem feeding herbivores (Walling, 2000; Pieterse *et al*., 2012; Erb & Reymond, 2019).

When plants are attacked by multiple herbivores, inhibition or potentiation between defence pathways may allow plants to fine tune defence responses to deal with a suite of herbivores (Li *et al*., 2018; Erb & Reymond, 2019). However, situations where crosstalk between pathways has an apparent negative effect for the plant may also occur (Koornneef & Pieterse, 2008; Thaler *et al*., 2012; Moreira *et al*., 2018). Phytohormonal crosstalk such as between SA and JA regulatory pathways may impair plant responses to one insect when the plant has already directed its resistance response towards a previous attacker of a different feeding guild (Pieterse *et al*., 2012; Soler *et al*., 2012; Moreira *et al*., 2018). To optimize resistance strategies to attack by a suite of herbivores, plants may have to reduce the costs of compromises in resistance to future attack when dealing with the curent attacker. Thus, plants may tailor plasticity in defence to arrival patterns of herbivores in the often species rich antagonist communities they are exposed to (Mertens *et al*., 2021). Yet, little research has addressed plant–herbivore interactions beyond dual attack. The few studies that included a third herbivore have identified that the order of attack by two herbivores and which species are involved in the interaction affect plant resistance to subsequent attack by a third herbivore (Mathur *et al*., 2013; Stam *et al*., 2014, 2017, 2018, 2019). Many plant species are attacked by an insect community that comprises more than three species, but how plants deal with their common situation of multi‐herbivore attack is poorly understood. A meriad of factors may be relevant for plant responses to multi‐herbivore attack. This includes the variation in herbivore traits such as feeding guild or food plant specialization, the order of arrival of herbivores and timing of herbivory during plant ontogeny. At the same time, diversity *per se* or components of diversity may affect species interactions (Loreau & Hector, 2011; Soliveres *et al*., 2016). Before disentangling the significance of each of these components, it should be established whether plants do respond differently to attack by a larger number of herbivore species than to a few and whether it matters that these species are more or less similar in traits.

In this study, we tested if species richness of previous attackers affects a plant’s capability to respond to a subsequent attacker of the same or of a different feeding guild. We hypothesized that an increasing richness of initial attackers leads to stronger feeding‐guild‐specific plant responses when all herbivores are of the same feeding guild than when plants are attacked by a mix of herbivores from different feeding guilds. The stronger response to a higher richness of attackers of the same feeding guild is expected to lead to increased resistance to subsequent attack by a herbivore of the same feeding guild, but to increased susceptibility to attack by a herbivore of a different feeding guild. To test these hypotheses, we subjected black mustard (*Brassica nigra*) plants to attack by an increasing richness (one, two or four species) of either phloem‐feeding, leaf‐chewing herbivores, or a mix of both. Over 20 different herbivore species may be found on *B. nigra* plants, but over their lifetime individual plants are on average colonized by eight different herbivore species out of this herbivore species pool (Mertens *et al*., 2021). We selected eight of the most prevalent herbivore species equally distributed across the leaf chewer and phloem feeder guilds and studied how plant responses to these attackers affect resistance to subsequent attack by caterpillars of the diamondback moth (*Plutella xylostella*). Caterpillars of *P. xylostella* typically attack *B. nigra* later in the season when plants have been previously attacked by other herbivores and virtually all plants in a stand are attacked by *P. xylostella* (Mertens *et al*., 2021). In addition, we quantified how combinations of these herbivores affect the expression of marker genes of the JA‐ and SA‐phytohormonal pathways to characterize induced plant resistance to multi‐herbivore attack. By identifying how plants deal with increased species richness of attack, we signal the importance of placing plant defence plasticity in the context of plant–insect community interactions.

## Materials and Methods

### Plants and insects

Three‐to‐four‐week‐old black mustard plants (*B. nigra*, Brassicales: Brassicaceae) were used for the experiments. Seeds were obtained from a natural population in the vicinity of Wageningen, the Netherlands (51°57′32′′N, 5°40′23′′E). The plants and the insects were cultured in a glasshouse at 22 ± 2°C, 60–70% relative humidity (RH) and 16 h : 8 h, L : D (light : dark) photoperiod regime. Eight insect species were used as primary herbivores or inducers of plant defence (Table 1). We used first‐instar larvae of herbivores of a leaf‐chewing feeding guild: the cabbage moth, *Mamestra brassicae* (*Mb*) (Lepidoptera: Noctuidae); the large cabbage white, *Pieris brassicae* (*Pb*) (Lepidoptera: Pieridae)*;* the turnip sawfly, *Athalia rosae* (*Ar*) (Hymenoptera: Tenthredinidae); and the mustard leaf beetle, *Phaedon cochleariae* (*Pc*) (Coleoptera: Chrysomelidae). For members of the phloem‐feeding guild we used the cabbage aphid, *Brevicoryne brassicae* (*Bb*)*;* the green peach aphid, *Myzus persicae* (*Mp*); the tobacco aphid, *Myzus persicae* sub. *nicotianae* (*Mpn*); and the mustard aphid, *Lipaphis erysimi* (*Le*) (all Hemiptera: Aphididae) (Table 1).

**Table 1:** Insects used for the experiment as inducers, in brackets abbreviations used throughout the document.

Species (abbreviation)	Picture	Order	Feeding guild
*Athalia rosae* (*Ar*)	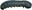	Hymenoptera	LC
*Mamestra brassicae* (*Mb*)	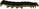	Lepidoptera	LC
*Phaedon cochleariae* (*Pc*)	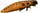	Coleoptera	LC
*Pieris brassicae* (*Pb*)	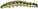	Lepidoptera	LC
*Brevicoryne brassicae* (*Bb*)	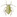	Hemiptera	PF
*Lipaphis erysimi* (*Le*)	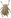	Hemiptera	PF
*Myzus persicae* (*Mp*)	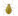	Hemiptera	PF
*Myzus persicae* sub*. nicotianae* (*Mpn*)	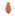	Hemiptera	PF

LC, Leaf chewer; PF, Phloem feeder.

As a subsequent herbivore (or receiver), second‐instar larvae of the diamondback moth *P. xylostella* (Lepidoptera: Plutellidae) were used. Larvae of this insect are specialists on brassicaceous plants and feed on foliar tissue, buds and flowers. *Mamestra brassicae*, *P. brassicae*, *B. brassicae* and *P. xylostella* were reared on Brussels sprouts plants (*Brassica oleracea* L. var. *gemmifera* cv Cyrus). *Myzus persicae*, *M. persicae* sub. *nicotianae*, *L. erysimi*, *A. rosae* and *P. cochleariae* were reared on radish (*Raphanus sativus*). All insects were obtained from the stock rearing of the Laboratory of Entomology, Wageningen University and were maintained at 22 ± 2°C, 60–70% RH and 16 h : 8 h, L : D photoperiod regime.

### Induced resistance to *P. xylostella* after multi‐herbivore attack

#### Species richness effect within feeding guilds

We first assessed how an increase in richness of attackers affects induced plant resistance to subsequent herbivory by *P. xylostella* caterpillars feeding on induced plants. Due to the large scale of our study and its associated constraints in terms of glasshouse space as well as numbers of caterpillars that can be weighed on a single day, we conducted separate experiments for leaf‐chewers (CHEW), phloem‐feeders (PHLO) and a mix of species of these feeding guilds (MIX). We thus focussed on quantifying the effect of species richness within each feeding guild.

We assessed plant responses to different levels of herbivore richness by infesting each plant with either one (leaf‐chewer: CHEW‐1; phloem‐feeder: PHLO‐1), two (CHEW‐2; PHLO‐2; MIX‐2) or four (CHEW‐4; PHLO‐4; MIX‐4) herbivore species while keeping total herbivore numbers constant. We excluded richness of three species, because of the imbalance that these treatments would have in number of leaf‐chewer and phloem‐feeder species. Each experiment included control plants that did not receive any inducing herbivore but were treated in a similar way as plants receiving herbivores (CON‐0). The total density used was four leaf chewers and eight phloem feeders for the leaf‐chewer and phloem‐feeder experiment respectively, and two leaf chewers plus four phloem feeders for the mixed richness experiment. We chose the number of species and the total density of inducers used based on field observations of insect communities of *B. nigra* plants (Poelman *et al*., 2009; Mertens *et al*., 2021). We allowed population growth of the initial infestation of the eight adult phloem feeders. For the mixed richness experiment, MIX‐2 resulted in 16 insect combinations of a single phloem‐feeder and leaf‐chewer, that were all tested. For MIX‐4 a selection of 12 of the 36 possible combinations of two phloem‐feeders and two leaf‐chewers were tested, selected to have the biggest differences between species combinations, while each herbivore is present the same number of times (six times) (Supporting Information Table S1). Thus, while testing the effect of leaf‐chewer richness we compared the performance of *P. xylostella* caterpillars on: (1) control plants (CON‐0), (2) plants induced by four larvae of a single chewer species (CHEW‐1, for each of the four chewer species), (3) plants induced by two larvae of two chewer species (CHEW‐2, for each of the six species combinations), (4) plants induced by four larvae, one of each of the four chewer species (CHEW‐4). For the phloem‐feeder richness experiment the setup was similar to the leaf‐chewer richness, with the only difference that the total number of inducer individuals was eight. For the mixed richness, we compared the performance of *P. xylostella* caterpillars on: (1) control plants (CON‐0), (2) plants induced by two larvae of one chewer species plus four phloem feeders of one species (MIX‐2, for each of the 16 species combinations), (3) plants induced by a single larva of two chewer species plus four phloem feeders (two of each species) (MIX‐4, for each of the 12 species combinations selected).

**Fig. 1:**
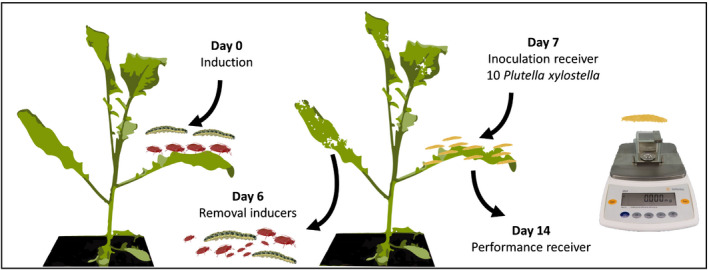
Experimental setup used to measure the performance of *Plutella xylostella* caterpillars on plants previously attacked by different species richness of phloem feeders, chewers or a mix of both (51 insect combinations).

Each experiment was divided into two temporal blocks. We prepared a total of 10 plant replicates (five per block) per insect combination for the leaf‐chewer and phloem‐feeder richness experiment, and eight plant replicates (four per block) per insect combination for the mixed richness experiment (Table 2). To prevent dehydration and cross‐contamination between treatments, 4–5 plants receiving the same herbivore combination were placed jointly in trays inundated with water. All treatments with unique species (combinations) replicating the species richness levels were randomized across the glasshouse. Insects were placed on the youngest fully expanded leaf of the plant and could freely feed from the plant for 1 wk (Fig. 1). Movement of herbivores between plants was prevented by water surrounding the pots in the inundated trays. The inducing herbivores were removed with a brush, after 7 d of feeding to exclude direct effects of inducing herbivores on the receiver herbivore. One day later, each plant was infested with 10 s instar *P. xylostella* larvae, acting as subsequent herbivore, or receiver. The mass of *P. xylostella* caterpillars was measured after 6 d of feeding on the control and induced plants as a proxy of plant resistance This was done by recapturing the *P. xylostella* larvae and weighing each individual on a Sartorius^®^‐CP2P‐Analytical Balance (accuracy 0.001 mg).

**Table 2:** Overview of treatments and replicates used for performance of *Plutella xylostella* experiment (approach 1) and for the experiment where we measured expression of defence related genes on *Brassica nigra* leaves.

Group	Diversity	Treatment	Performance			Gene expression	
			Plant replicates	Trays	Replicates group	Biological replicates	Replicates group
Control	0	Ctrl	10	2	10	5	5
Leaf chewers 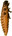	1	*Mb*	10	2	40	5	20
		*Ar*	10	2		5	
		*Pb*	10	2		5	
		*Pc*	10	2		5	
	2	*Mb–Ar*	10	2	60	5	30
		*Mb–Pb*	10	2		5	
		*Mb–Pc*	10	2		5	
		*Ar–Pb*	10	2		5	
		*Ar–Pc*	10	2		5	
		*Pb–Pc*	10	2		5	
	4	*Mb–Ar–Pb–Pc*	60	12	60	20	20
Phloem feeders 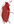	1	*Bb*	10	2	40	5	20
		*Mp*	10	2		5	
		*Mpn*	10	2		5	
		*Le*	10	2		5	
	2	*Bb–Mp*	10	2	60	5	30
		*Bb–Mpn*	10	2		5	
		*Bb–Le*	10	2		5	
		*Mp–Mpn*	10	2		5	
		*Mp–Le*	10	2		5	
		*Mpn–Le*	10	2		5	
	4	*Bb–Mp–Mpn–Le*	60	12	60	20	20
Mix (leaf chewers + phloem feeders) 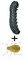	2	*Bb–Mb*	8	2	128	2	32
		*Bb–Ar*	8	2		2	
		*Bb–Pb*	8	2		2	
		*Bb–Pc*	8	2		2	
		*Mp–Mb*	8	2		2	
		*Mp–Ar*	8	2		2	
		*Mp–Pb*	8	2		2	
		*Mp–Pc*	8	2		2	
		*Mpn–Mb*	8	2		2	
		*Mpn–Ar*	8	2		2	
		*Mpn–Pb*	8	2		2	
		*Mpn–Pc*	8	2		2	
		*Le–Mb*	8	2		2	
		*Le–Ar*	8	2		2	
		*Le–Pb*	8	2		2	
		*Le–Pc*	8	2		2	
	4	*Bb–Mp–Mb–Ar*	8	2	96	2	24
		*Bb–Mp–Pb–Pc*	8	2		2	
		*Bb–Mpn–Mb–Pc*	8	2		2	
		*Bb–Mpn–Ar–Pb*	8	2		2	
		*Bb–Le–Mb–Pb*	8	2		2	
		*Bb–Le–Ar–Pc*	8	2		2	
		*Mp–Mpn–Mb–Pb*	8	2		2	
		*Mp–Mpn–Ar–Pc*	8	2		2	
		*Mp–Le–Mb–Pc*	8	2		2	
		*Mp–Le–Ar–Pb*	8	2		2	
		*Mpn–Le–Mb–Ar*	8	2		2	
		*Mpn–Le–Pb–Pc*	8	2		2	

We show replicates per treatment and per species richness within each group (leaf chewer, phloem feeder, mix). *Plutella xylostella* performance was measured in three separate experiments (leaf chewer, aphid, mix), each of them divided in two blocks over time. Each of these blocks contained five control plants (species richness 0, no insects). The replicates shown are the total of replicates for the two blocks together. Each replicate consisted of one *Brassica nigra* plant where we inoculated 10 *P. xylostella*. The gene expression analysis was done in a single experiment. Here, each biological replicate consisted of a pool of leaf discs from three plants. We prepared a set of biological replicates that were sampled 48 h after induction, and another set that was sampled at 96 h after induction. Each plant was sampled only once. Leaf chewers: *Mb*,* Mamestra brassicae*; *Ar*, *Athalia rosae*; *Pb*,* Pieris brassicae*; *Pc*,* Phaedon cochleariae*. Aphids: *Bb*,* Brevicoryne brassicae*; *Mp*,* Myzus persicae*; *Mpn*,* Myzus persicae* sub. *nicotianae*; *Le*,* Lipaphis erysismi*.

#### Comparing direction of effects by feeding guilds

After establishing in separate experiments for feeding guild the effect of species richness within feeding guild on the performance of *P. xylostella* and in which direction each feeding guild affected *P. xylostella* performance, we performed an additional experiment to directly compare the effect of feeding guild on *P. xylostella* performance. We selected the most extreme treatments of each main experiment: CON‐0 (control), phloem‐feeder (PHLO‐4), leaf‐chewer (CHEW‐4) and mixed (MIX‐4) and prepared 24 plant replicates per treatment (Table S2). We randomized individual plants over the glasshouse and followed the same protocol of inducing the plants, removing the herbivores used for induction and assessing the performance of *P. xylostella* caterpillars on these plants. All performance experiments were performed in the same glasshouse compartment (December 2017 to July 2018) (19°C, 60–70% RH and 16 h : 8 h, L : D photoperiod regime).

### Analysis of *B. nigra* gene expression

We characterized *B. nigra* physiological responses to attack by the herbivore combinations in a single separate experiment (same treatments used for measuring performance, Table 2). The experiment was conducted in a different glasshouse compartment than the performance tests, but with similar climate conditions (July–August 2018) (19°C, 60–70% RH and 16 h : 8 h, L : D photoperiod regime). To characterize plant responses, we selected homologues of genes that are known to regulate JA‐ and SA‐plant responses to insect‐herbivore attack. We analysed expression levels of the JA‐biosynthesis gene *LIPOXYGENASE 2* (*LOX2*), the JA‐responsive gene *VEGETATIVE STORAGE PROTEIN2* (*VSP2*), the SA‐biosynthesis gene *ISOCHORISMATE SYNTHASE* (*ICS*), the SA‐responsive gene *PATHOGENESIS‐RELATED PROTEIN 1* (*PR1*) and the crosstalk gene *WRKY70*. Biosynthesis of the lipid‐derived phytohormone JA starts when injury of plant cells releases α‐linolenic acid from the plastid membranes (Wasternack & Song, 2017). A key regulator of the first steps in JA biosynthesis is the enzyme lipoxygenase (LOX) which activates expression of JA‐biosynthetic genes, such as *LOX2* (Bell *et al*., 1995). The bioactive form of JA (JA‐Ile) is synthesized after a few enzymatic steps and transported to the nucleus. JA‐Ile triggers the degradation of the *JASMONATE‐ZIM DOMAIN* (*JAZ*), a repressor of JA biosynthesis. JAZ degradation releases repression of MYC transcription factors, resulting in expression of JA‐responsive genes, such as *VSP2* (Erb & Reymond, 2019). SA can be synthesized via the *PHENYLALANINE AMMONIA LYASE* (PAL) and the *ISOCHORISMATE SYNTHASE* (ICS) enzymatic pathways (Pieterse *et al*., 2012). Signalling downstream of SA biosynthesis is regulated by transcription factors that activate expression of pathogenesis‐related defence genes (*PR* genes) (Pieterse *et al*., 2012). Additionally, WRKY transcription factors play a key role in SA signalling, as they activate or repress SA responses (Li *et al*., 2004).

We focussed on characterizing how plants respond to attack when herbivores are coming from the same feeding guild (phloem‐feeders or leaf‐chewers) or from multiple feeding guilds. Within feeding guild, we then addressed whether individual herbivores, number of herbivore species and specific combinations differ in induced plant responses. Gene expression in response to herbivory was assessed at 48 and 96 h after induction, using a different set of plants per time point so that plants were only sampled once. Our earlier experiments with *B. nigra* identified that with the two time points the expression patterns of the selected genes can be well characterized (Mertens *et al*., 2021). We prepared five biological replicates per treatment for control (CON‐0), leaf‐chewer (CHEW‐1; for each of the four leaf‐chewer species) and phloem‐feeder (PHLO‐1; for each of the four phloem‐feeder species) and five replicates for each CHEW‐2 and PHLO‐2 species combination (six species combinations for leaf chewers and phloem feeders) (Table 2). We prepared 20 biological replicates for treatments with four herbivore species CHEW‐4 and PHLO‐4 to balance the number of replicates across species richness treatments. MIX‐2 (16 species combinations) and MIX‐4 (12 species combinations) had two biological replicates per species combination, which resulted in 32 biological replicates for MIX‐2 and 24 for MIX‐4, respectively. Each biological replicate contained a leaf disc (ø = 2 cm) that was taken with a sterilized puncher from three plants with the same treatment, sampling each plant only once. Leaf samples were placed in an Eppendorf tube, immediately flash frozen in liquid nitrogen and stored at −80°C until further analysis (Fig. 1). Induction of plants with the herbivore treatments was performed on the youngest fully‐expanded leaf. Directly after induction, the induced leaf was enclosed in a mesh bag to confine herbivores to a single leaf. Control plants received a mesh bag without insects. Biological replicates represented by the group of three plants that would be combined in a single sample were randomly placed in the glasshouse.

### RNA extraction and PCR protocol

The frozen samples were ground to a fine powder with a sterile pestle. This was followed by RNA extraction using the Qiagen RNeasy 96 Kit (Venlo, the Netherlands) with an adjusted protocol for plants. Briefly, 450 µl of RTL buffer were added to each Eppendorf tube (which contained approximately 50 mg of frozen, ground leaf sample). Samples were incubated at 56°C for 1 to 3 min and were centrifuged at 10 000 ***g***, until a tight pellet was formed (8–10 min). The supernatant was transferred to new Eppendorf tubes and mixed with 0.5 volumes of 96% ethanol. Each sample was transferred to a 96‐RNeasy plate and that was centrifuged 6 min at 3700 ***g***. The flow‐through was discarded, and each well was treated with 80 µl of a solution of DNase I in RDN buffer (1 : 7) which was added to the tube directly onto each membrane and was incubated for 15 min at room temperature. To each sample, 800 µl of RWI buffer were added, and after 5 min of incubation the plate was centrifuged for 6 min at 3700 ***g*** and the flow‐through was discarded. Now, 800 µl of RPE buffer were added to each sample, and the plate was centrifuged for 6 min at 3700 ***g***, the flow‐through was discarded and the plate was centrifuged for 10 min at 3700 ***g***. The plate was placed on a rack of collection microtubes, where 45 µl of RNAse free water (Qiagen) were added to each sample and after 1‐min incubation, the plate was centrifuged for 6 min at 3700 ***g***. The RNA concentration was measured using a DS‐II FX Spectrophotometer/Fluometer (DeNoVix, Wilmington, DE, USA). Samples were diluted and adjusted to an RNA concentration of 66.6 ng μl^−1^. From the RNA samples complementary DNA (cDNA) was synthesized, using the SensiFAST cDNA synthesis kit (Bioline, Memphis, TN, USA). We quantified the expression levels of the target genes *LOX2*, *VSP2*, *ICS* and *PR1* and of the reference genes *GAPDH* and *SAR1A* by quantitative polymerase chain reaction (qPCR) using the SensiFAST SYBR no‐ROX kit (Bioline). We added 5 ng of the cDNA template to the reaction with a total volume of 20 µl. The reactions were performed using a CFX96 Touch™ Real‐Time PCR Detection System (Bio‐Rad, Hercules, CA, USA). All reactions were conducted using two technical replicates and samples were omitted from further analyses if the difference in expression of technical replicates was higher than 0.5 (4% of the samples). Plate setups included negative controls (no template) and inter‐run calibrators.

We tested the following reference genes: *ACTIN‐2* (*ACT2*)*, BETA‐TUBULINE* (*B‐TUB*)*, ELONGATION FACTOR‐1* (*EF1*)*, PEROXIDASE 4* (*PER4*)*, SECRETION ASSOCIATED RAS RELATED GTPASE 1A* (*SAR1A*)*, GLYCERALDEHYDE‐3‐PHOSPHATE DEHYDROGENASE* (*GAPDH*). The last two reference genes were selected because they had highest expression stability regardless of the treatment (primer sequences are presented in Table S3). We used the expression of the reference genes to calculate the relative expression of genes of interest in each sample subjected to the different treatments.

### Statistical analysis

#### Performance data

We first analysed for each of the feeding guilds separately how species richness of inducing herbivores affected plant resistance to the subsequent feeding by *P. xylostella* caterpillars. The performance data (weight of individual *P. xylostella* caterpillars) was cube‐root transformed (third level root transformation) to better approach the assumptions of normality and constant variance. We fitted Mixed Linear Models (MLMs), using a fixed effect for species richness with the levels of richness 1, 2 and 4 for the model on phloem feeders and the model for leaf‐chewers, the levels 2 and 4 for the model of the mixed feeding guilds. We included the sub‐treatments of each of the unique species or species combinations (11 for phloem feeders, 11 for leaf chewers, and 28 for mix). We present the outcomes of these models with two sets of random effects (Table S4). One set of models that included the random structure for blocks, trays within blocks, plants within trays, and residual error. The second set of models excluded the tray effect from the random structure. Even though in this particular statistical approach the random factor ‘tray’ unexpectedly explained a substantial amount of variation, we purposely removed this factor from the model. The tray corresponded with the variation of sub‐treatments of species combinations within the richness levels and likely at least to an extend contains the biological variation caused by unique species combinations. We kept a smaller scale random factor of plant, and we aimed to test the biological effect of the sub‐treatments within each main experiment (species included in phloem‐feeder, leaf‐chewer and mixed treatments) represented by trays with sub‐treatments randomly distributed in the glasshouse. Based upon the fitted model, by averaging over the insect combinations that constituted a richness level within guild, and combining the estimated means linearly, we estimated linear regression lines for species richness within guild, using facilities for user‐defined linear combinations of the software. Next, we tested nullity of slopes within guilds to identify whether there was a significant positive or negative correlation between species richness of inducers and performance of *P. xylostella* (slopes excluded performance of caterpillars on undamaged plants). Besides linear trends, also quadratic trends were tested. Since both analyses gave similar results, we present only linear trends in the results section. With the Mixed Model, we then tested for significance of the different richness levels with performance of the caterpillars on undamaged control plants. These analyses were followed by *post hoc* analyses to compare individual sub‐treatments. Finally, we compared the slopes for the three feeding guilds obtained in the separate experiments among each other to identify whether relationships between species richness and *P. xylostella* performance differed for the feeding guilds. Because this was an indirect comparison combining data obtained at different moments, we had performed an additional experiment that directly compared performance of *P. xylostella* on undamaged control plants, and the most extreme richness of phloem‐feeders, leaf‐chewers or a mix of feeding guilds. Here individual plants were placed randomly distributed in the glasshouse and a single block was performed. These data were analysed with a Mixed Model for the fixed treatment effect feeding guild (CON‐0, PHLO‐4, CHEW‐4, MIX‐4). For the random part, besides the residual variance, only random effects for plants were included.

Statistical analysis for herbivore performance was done in R v.3.6.1 (R Core Team, 2019) and using proc mixed of Sas (v.6.4). For all hypothesis tests we used 0.05 as level of significance.

#### Gene expression

Gene‐expression data was imported to Biogazelle qbase + 3.1 (Zwijnaarde, Belgium) to calculate the relative gene expression level between different samples for a given target gene (*LOX2*, *VSP2*, *ICS*, *PR1*, *WRKY70*) corrected by the expression value of the reference genes (*GAPDH*, *SAR1A*). Data was corrected for differences between runs using inter‐run calibrators and data was Log_10_ transformed to meet the assumptions of the model. For each of the five genes and separately per time point, we ran General Linear Models (GLMs) with *post hoc* test (least significant difference (LSD)) in R v.3.6.1 (R Core Team, 2019). We first tested whether the nine different treatment groups based on the combination of feeding guild and species richness differed in expression levels. These analyses identified that expression patterns were strongly determined by feeding guild. We then deepened the analysis by testing in separate models per feeding guild whether the species richness levels significantly affected gene expression, and used *post hoc* analyses to compare individual richness levels within feeding guild as well as individual sub‐treatments represented by the unique herbivore combinations within feeding guild.

## Results

### Species richness affects induced plant resistance

**Fig. 2:**
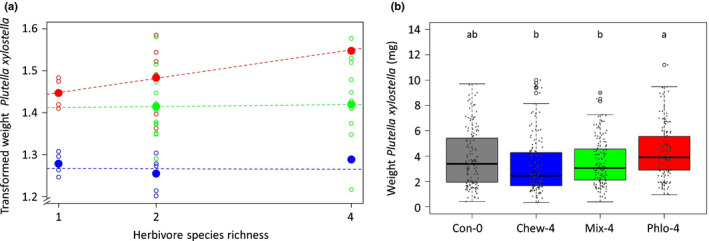
(a) Regression line of the transformed performance of *Plutella xylostella* on *Brassica nigra* plants. Performance of caterpillars in different experimental blocks is presented in weight corrected to the average weight of *P. xylostella* on untreated control plants across all blocks. Plants were treated with an increasing richness (one, two or four species) of phloem feeders (red), leaf chewers (blue), or a mix of both (green), or were left untreated. The open circles represent averages within each sub‐treatment (i.e. insect combination). The phloem‐feeder and leaf‐chewer experiments contained four sub‐treatments for species richness 1, six sub‐treatments for species richness 2 and one sub‐treatment for species richness 4. The mixed species richness experiment contained 16 sub‐treatments for mixed species richness 2 and 12 sub‐treatments for mixed species richness 4. The closed circles represent averages of the sub‐treatments within each species richness level. Lighter‐coloured circles show treatments that only received one herbivore species, middle‐dark‐coloured circles show treatments that received two herbivore species, darker‐coloured circles show treatments that received four herbivore species. (b) Performance of *P. xylostella* on approach 2: in one experiment, plants were treated with four species of phloem feeders (PHLO‐4), leaf chewers (CHEW‐4), a mix of both (MIX‐4) or left untreated (CON‐0) (*n* = 24). Boxplot height corresponds to the first and third quartiles (Q1 and Q3), and the middle line to the median. Letters above the boxplots show significant differences (MLM, *post hoc* Tukey).

**Fig. 3:**
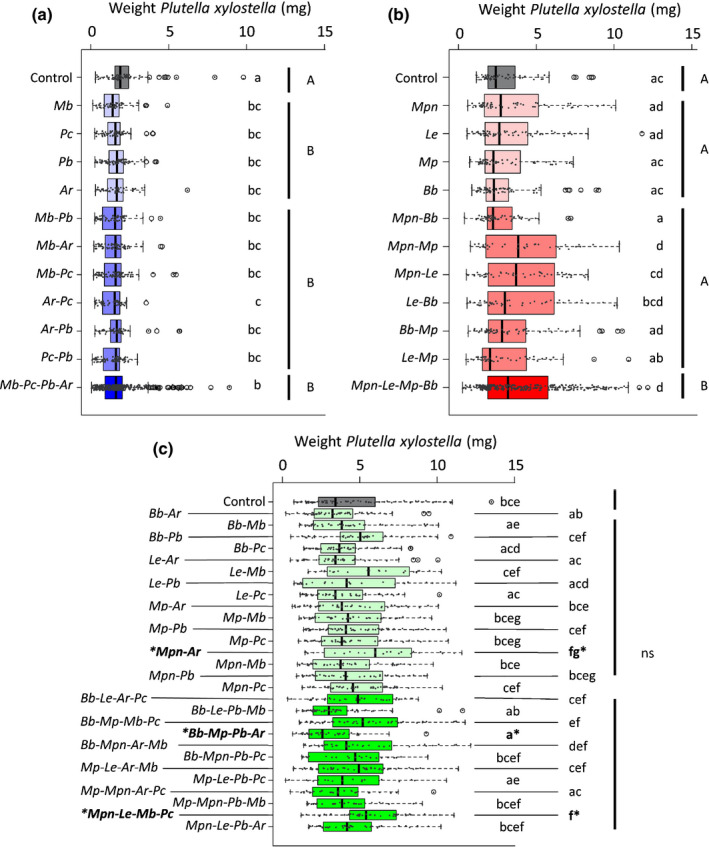
Weight (mg) of *Plutella xylostella* larvae after feeding for 6 d from *Brassica nigra* plants previously attacked by leaf chewers (blue), phloem feeders (red), a mix of both (green), or from untreated plants (grey, control). Panels (a–c) show the performance of *P. xylostella* in approach 1, where we measured performance in three separate experiments. Lighter boxplots show treatments that only received one herbivore species, middle‐dark boxplots show treatments that received two herbivore species, darker boxplots show treatments that received four herbivore species. (a) Performance of *P. xylostella* on plants previously attacked by leaf chewers (*n* = 10). (b) Performance of *P. xylostella* on plants previously attacked by phloem feeders (*n* = 10) (c) Performance of *P. xylostella* on plants previously attacked by a mix of leaf chewers and phloem feeders (*n* = 10 for control, *n* = 8 for the rest). Combination of inducers that affected *P. xylostella* growth (compared to control, untreated plants) are marked in bold and with an asterisk. Boxplot height corresponds to the first and third quartiles (Q1 and Q3), and the middle line to the median. Letters above the boxplots show significant differences (MLM, *post hoc* Tukey). Letters for (c) can be found in Supporting Information Table S4. ns, not significant. Leaf chewers: *Mamestra brassicae* (*Mb*), *Phaedon cochleariae* (*Pc*), *Pieris brassicae* (*Pb*), *Athalia rosae* (*Ar*). Phloem‐feeding aphids: *Myzus persicae* sub. *nicotianae* (*Mpn*), *Lipaphis erysimi* (*Le*), *Myzus persicae* (*Mp*), *Brevicoryne brassicae* (*Bb*).

Plants attacked by a higher species richness of phloem feeders became more susceptible to caterpillars of the subsequent attacker *P. xylostella* as indicated by an increased caterpillar performance on plants induced by larger number of aphid species (significant positive slope of species richness 1, 2 or 4, with tray: *P* = 0.095; without tray *P* < 0.01; Fig. 2a). A richness of four aphid species resulted in significant susceptibility to *P. xylostella* compared to its performance on undamaged control plants and susceptibility increased when four species induced plants compared to one or two aphid species (Mixed Model; without tray *F*_3,438_ = 4.66; *P* = 0.0032) (Fig. 3a). Individual attack by each of the four phloem‐feeding species only slightly enhanced performance of *P. xylostella* to levels not significantly different from performance on undamaged plants (Fig. 3b). However, the specific combination of initial attack by the aphid species *M. persicae* sub. *nicotianae*–*M. persicae*, *M. persicae* sub. *nicotianae*–*L*. *erysismi* or *L*. *erysismi*–*B*. *brassicae* significantly enhanced performance of *P. xylostella* caterpillars (Fig. 3b).

By contrast, a higher species richness of leaf‐chewers did not lead to effects on resistance to *P. xylostella* (no significant slope of species richness 1, 2 or 4; with tray *P* = 0.97; without tray *P* = 0.97; Fig. 2a). Plant induced responses to feeding by one, two or four species of leaf‐chewers equally enhanced resistance to *P. xylostella* caterpillars as indicated by decreased performance of these caterpillars compared to their performance on undamaged control plants (Mixed Model; without tray *F*_3,248_ = 5.39; *P* = 0.013) (Fig. 3b). All four leaf‐chewing herbivores reduced *P. xylostella* performance similarly when attacking the plant as individual species (Fig. 3a). The combination of the leaf chewers *A. rosae* and *P. cochleariae* had the strongest negative effect on the performance of *P. xylostella* caterpillars, but did not differ significantly from other treatments with two leaf‐chewer species (CHEW‐2) or plants damaged by single chewer species (CHEW‐1) or the full set of four species (CHEW‐4) (Fig. 3a). Induction of plants by a mix of phloem‐feeding and leaf‐chewing herbivores with either two or four different species did not affect the performance of *P. xylostella* caterpillars (slope for richness 2 and 4 not different from 0: with tray *P* = 0.81; without tray *P* = 0.81) (Fig. 2a) and on average the performance of *P. xylostella* on plants induced with a mix of feeding guilds was not different from its performance on undamaged control plants (Mixed Model; without tray *F*_2,485_ = 0.03; *P* = 0.97) (Fig. 3c). Despite these overall neutral effects, plants previously attacked by a specific combination of *M. persicae* sub. *nicotianae*–*A. rosae* or a mix of the four species *M. persicae* sub. *nicotianae*–*L. erysimi*–*M. brassicae*–*P. cochleariae* became less resistant to larvae of *P. xylostella* (Table S5; Fig. 3c). By contrast, plants previously attacked by a mix of the four species *B. brassicae*–*M. persicae*–*P. brassicae*–*A. rosae* became more resistant to larvae of *P. xylostella* (Table S5; Fig. 3c).

In our second independent experiment where individual plants could be randomly distributed in the glasshouse, we confirmed that performance of *P. xylostella* caterpillars differed for plants induced by phloem‐feeders, leaf‐chewers or a mix of herbivores from different feeding guilds (Mixed Model, *F*_3,77_ = 4.37, *P* = 0.0068) (Fig. 2b). Direct comparison of *P. xylostella* performance on plants induced by species richness of four herbivores supported that phloem‐feeders increased performance of *P. xylostella* caterpillars and differed from the reduced performance of caterpillars feeding on leaf‐chewer induced plants (Fig. 2b). Caterpillars feeding from plants induced by a mix of feeding guilds had intermediate performance and had significantly lower average mass compared to caterpillars feeding on phloem‐feeding induced plants (Fig. 2b). Indirect comparison of the direction of slopes in the first series of experiments further supports that the direction of effect of species richness differs between phloem‐feeders and the slope of leaf‐chewers or the mixed herbivore treatments (PHLO vs CHEW with tray *P* = 0.22, without tray *P* < 0.05; PHLO vs MIX with tray *P* = 0.24, without tray *P* < 0.05; CHEW vs MIX with tray *P* = 0.86, without tray *P* = 0.84).

To summarize, species richness of initial attackers as well as species composition across feeding guilds affects induced plant resistance to subsequent herbivory. Plants attacked by phloem‐feeding insects became more vulnerable to larvae of *P. xylostella*, whereas plants attacked by leaf chewers became more resistant to *P. xylostella* caterpillars. Species richness of phloem feeders was more important in affecting *P. xylostella* performance than richness of leaf chewers. For leaf‐chewers the presence of a single herbivore species caused similar effects compared to four species of leaf‐chewing herbivores. *Plutella xylostella* caterpillars performed equally well on plants induced with a mix of herbivore species from the two feeding guilds regardless of species richness and on plants that had not been exposed to herbivores.

### Feeding guild specific induced gene expression

**Fig. 4:**
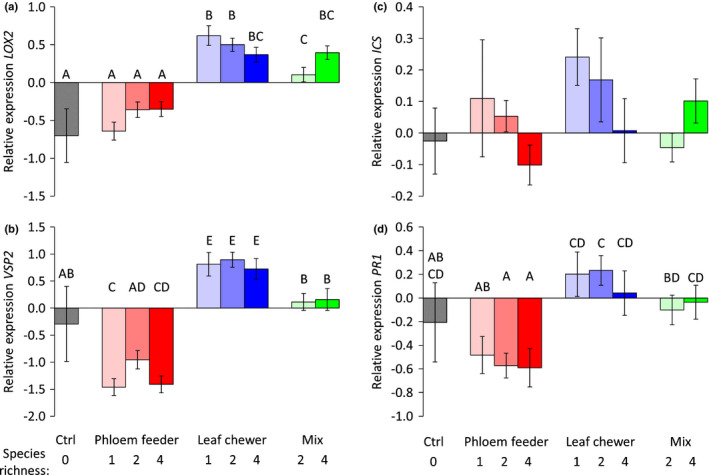
Relative gene expression of *Brassica nigra* leaves at 48 h after infesting them with an increasing species richness (one, two or four species) of aphids, chewers, a mix of both or untreated plants (Ctrl). We measured the expression of JA‐biosynthesis and JA‐responsive genes *LOX2* and *VSP2* (a) and (b), and the SA‐biosynthesis and SA‐responsive genes *ICS* and *PR1* (c) and (d). Bars represent mean ± SE of log transformed data. Gene expression is relative to the expression level of two reference genes *GAPDH* and *SAR1A*. Bars not sharing letters are significantly different from each other (LM, *post hoc* LSD).

*Brassica nigra* attacked during 48 and 96 h by leaf chewers, or by a mix of leaf chewers and phloem feeders showed induced expression of the JA‐biosynthesis and JA‐responsive genes (*LOX2* and *VSP2,* respectively) and of the SA‐responsive gene (*PR1*) (Linear Model (LM) 48 h: *LOX2* *F*_8,149_ = 20.58, *P* < 0.001; *VSP2* *F*_8,149_ = 24.93, *P* < 0.001; *PR1* *F*_8,149_ = 4.64, *P* < 0.001, Fig. 4; LM 96h: *LOX2* *F*_8,149_ = 43.70, *P* < 0.001; *VSP2* *F*_8,149_ = 18.0, *P* < 0.001; *PR1* *F*_8,149_ = 3.51, *P* < 0.001, Supporting Information Fig. S1). By contrast, phloem feeders did not induce significant expression of these genes compared to undamaged control plants. None of the herbivore treatments significantly affected expression of the SA‐biosynthesis gene *ICS* and of the crosstalk gene *WRKY70* at either 48 or 96 h of herbivore attack (LM, 48 h: *ICS* *F*_8,149_ = 1.0, *P* = 0.44; *WRKY70* *F*_8,149_ = 1.35 *P* = 0.22, Fig. 4; LM 96 h: *ICS* *F*_8,149_ = 1.08, *P* = 0.38; *WRKY70* *F*_8,149_ = 1.12 *P* = 0.30, Fig. S1). 48 and 96 h of feeding by the leaf chewers and by each of the leaf‐chewer species combinations induced the JA‐biosynthesis gene *LOX2* (Figs S2, S3). In addition to the direction of plant responses driven by feeding guild, within feeding guilds species richness affected gene expression only for leaf‐chewing herbivores (Figs S2–S5). For leaf‐chewing herbivores, 96 h of feeding by a combination of two herbivores enhanced gene expression of *LOX2* (*F* = 12.16, *P* < 0.001) and *VSP2* (*F* = 4.54, *P* < 0.01) compared to single herbivore attack (Fig. S3). Herbivory by four species could not be discriminated in gene expression patterns from those induced by one or two leaf‐chewing herbivores (Fig. S3). Individual leaf‐chewer species and combinations of specific leaf chewers differed in the magnitude of induced expression of *LOX2* (Fig. S3a). Plants attacked by *P. cochleariae* or by *A. rosae* had stronger *LOX2* induction than plants attacked by *M. brassicae*. Feeding by the combinations of *A. rosae*–*P. cochleariae*, *A. rosae*–*P. brassicae* or *P. brassicae*–*P. cochleariae* induced the highest expression levels of *LOX2* (Fig. S3a). Similarly, most of the leaf chewers (combinations) induced the JA‐responsive gene *VSP2* with the only exception of plants attacked by *M. brassicae*, in which expression of *VSP2* was not different from control plants. The combination of *P. brassicae*–*P. cochleariae* caused the strongest induction of *VSP2* at 96 h (Fig. S3b). The SA‐responsive gene *PR1* was induced by attack of some (combination of) leaf chewers (Fig. S3d). Single attack by *P. cochleariae* or *A. rosae*, dual attack by *M. brassicae*–*A. rosae*, *M. brassicae*–*P. cochleariae*, or *A. rosae*–*P. cochleariae* and feeding by the four leaf‐chewers induced expression of *PR1* at 96 h (Fig. S4d). By contrast, feeding by phloem feeders and by each of the phloem‐feeder species combinations did not induce any of the defence marker genes tested at 48 or 96 h after attack (Figs S4, S5), except for *M. persicae* that induced *LOX2* expression 48 h after feeding (Fig. S4).

In summary, feeding by the leaf chewers individually or in combination, induced the expression of the JA‐biosynthesis and JA responsive genes *LOX2* and *VSP2*, respectively. Feeding by a mix of leaf‐chewing and phloem‐feeding insects, also induced *LOX2* and *VSP2*, but to a lesser extent. Feeding by the phloem feeders, or by combination of phloem feeders did not induce any of the marker genes tested but repressed the expression of the JA‐responsive gene *VSP2* 48 h after attack.

## Discussion

Multi‐herbivore attack, species richness of herbivore attack as well as the composition of the species across feeding guilds influenced induced plant responses and resistance to subsequent herbivory. Plant responses to phloem‐feeding insects promoted the growth of *P. xylostella*, whereas plant responses to leaf chewers reduced the performance of *P. xylostella* caterpillars. The higher the species richness of phloem feeders attacking the plant, the more vulnerable the plant became to larvae of the subsequent attacker *P. xylostella*. By contrast, induction by any combination of leaf‐chewers regardless of species richness led to stronger resistance to *P. xylostella*. When the plant was attacked by a mix of phloem feeders and leaf chewers *P. xylostella* caterpillars performed equally well on plants induced with a mix of herbivore species from the two feeding guilds and on plants that had not received initial herbivore attack. Unexpectedly, in our study, the factor tray explained a substantial amount of variation. Plants sharing the same tray, were not only receiving the same herbivore, but could have affected each‐other more strongly than plants in neighbouring trays through volatile or root communication via the shared pool of water. The compromise of plants in trays we had to make for feasibility of the experiment confounded biological effects associated with trays and those on our treatment level. Because trays were randomly distributed in the glasshouse having different neighbouring treatments, we deem it unlikely that volatile or root communication drives the patterns observed in our experiments. By using multiple species combinations per species richness level that were randomly placed in the glasshouse, independent blocks and experiments that all present similar findings, the most likely conclusion is the biological effect of species richness and feeding guild of the herbivores driving plant responses and resistance to *P. xylostella*.

The stronger susceptibility to larvae of *P. xylostella* after induction by a richer phloem feeder community suggests an additive effect of each phloem feeder species on suppressing plant responses to a leaf chewer. When feeding from the phloem sap, aphids inject effectors (i.e. aphid salivary proteins) that alter the plant primary and secondary metabolism (Mutti *et al*., 2008; Giordanengo *et al*., 2010; Elzinga & Jander, 2013; Züst & Agrawal, 2016; Ahman *et al*., 2019; Jiang *et al*., 2019). Excretion of salivary effectors by *M. persicae* has been found to reduce plant secondary metabolite production and callose deposition, benefitting aphid population growth (Elzinga *et al*., 2014). The enhanced performance of *P. xylostella* on plants previously attacked by a higher species richness of phloem feeders, could potentially be attributed to a higher diversity of salivary effectors with more aphid species increasing the strength of the antagonistic effect of SA regulated resistance on JA responses. In our study, aphid feeding suppressed the expression of the JA‐responsive gene *VSP2* 48 h after attack. The JA pathway is a well‐known defence pathway against insect herbivores and thus JA suppression is a potential explanation of the increased growth of *P. xylostella* on aphid‐induced plants (Zarate *et al*., 2007; Onkokesung *et al*., 2010). The JA suppression, might be attributed to the aphids manipulating the host plant (Will *et al*., 2007; Ahman *et al*., 2019). However, we did not detect an aphid‐induced expression of any of the two SA‐marker genes studied nor the SA/JA crosstalk gene *WRKY70*. Thus, we do not have support to explain the aphid‐induced changes based on the SA/JA negative crosstalk paradigm (Kroes *et al*., 2015; Onkokesung *et al*., 2016). Further research is needed to unravel the mechanisms behind the aphid‐induced facilitation of the growth of *P. xylostella*.

In contrast with the phloem‐feeder‐induced vulnerability to *P. xylostella*, the growth of *P. xylostella* larvae was negatively affected by previous chewer attack with no role for species richness. Attack by a single leaf‐chewer had a similar reduction on *P. xylostella* performance compared with treatments that were richer in leaf‐chewer species. The absence of effect caused by species richness of chewers compared to the effect of richness caused by phloem feeders contrasts with the higher taxonomic richness used in the chewers than in the phloem feeders (all aphids). Thus, even with a wider phylogenetic richness of chewer species, combining these species did not strongly affect plant responses to chewing herbivores. Also in research on lima bean (*Phaseolus lunatus*), induced plant responses were not herbivore‐specific and simultaneous attack by two chewers had a similar effect compared to single attack (Moreira *et al*., 2015). On wild radish plants (*Raphanus raphanistrum* and *R. sativus*) previously attacked by *P. xylostella* or *Spodoptera exigua* became more resistant to larvae of *S. exigua, Trichoplusia ni* (cabbage looper) and *Pieris rapae* (small cabbage butterfly) (Agrawal, 1999, 2000; Williams *et al*., 2005; Rodriguez‐Saona *et al*., 2010). Interestingly in the study on wild radish, the strength and direction of the plant‐mediated effect of chewer on chewer depended on the species involved, as previous infestation by *P. rapae* induced resistance only to *S. exigua* and *P. rapae*, and previous damage by *T. ni* (generalist) did not affect the performance of any of the herbivores (Agrawal, 2000). The fact that all leaf chewers and their combinations in our study equally affected plant resistance to a subsequent attacker only partly matches our molecular analysis, where we found that all chewers (and all chewer combinations) induce the JA‐biosynthesis and JA‐responsive genes *LOX2* and *VSP2* at the two time‐points measured. However, single leaf chewer species induced these genes less strongly than a combination of two leaf chewer species. In *Arabidopsis thaliana,* leaf chewers had a strong induction of the JA‐responsive gene *VSP2*, and also became more resistant to subsequent chewers (Rasmann *et al*., 2015). Additionally, we found that feeding by chewers induced expression of the SA‐responsive gene *PR1*. Similarly, other studies reported induction of both SA and JA pathways upon leaf chewer feeding (Heidel & Baldwin, 2004; Li *et al*., 2016, 2016a,b; Arena *et al*., 2018; Kielkiewicz *et al*., 2019). In *Brassica* interactions with chewing insects, specificty in response to leaf chewing herbivores may be low and suggests a less important role of Herbivore Associated Molecular Patterns (HAMP) and herbivore specific effectors than found for other plant–insect interactions (Erb & Reymond, 2019).

Plants simultaneously attacked by leaf chewers plus phloem feeders show similar resistance to larvae of *P. xylostella* compared to plants that did not receive herbivory. This suggests that the effect of aphid‐induced vulnerability cancels out the effect of chewer‐induced resistance to larvae of *P. xylostella*. In line with our findings, cotton plants simultaneously attacked by the chewer *S. exigua* and by the phloem feeder *Bemisia tabaci* emitted lower amounts of herbivore‐induced plant volatiles than plants that were only attacked by *S. exigua* (Rodriguez‐Saona *et al*., 2003). Likewise, *S. exigua* oviposition was deterred on tomato plants damaged by chewers, but the deterrence disappeared on plants that were simultaneously damaged by aphids and chewers (Rodriguez‐Saona *et al*., 2005). In our study, the expression of the two JA‐related genes investigated was lower on plants simultaneously attacked by aphids and chewers compared to plants only under chewer attack. Similarly, caterpillar feeding on milkweed (*Asclepias syriaca*) induced JA, cardenolides and latex, and this induction was substantially attenuated in the presence of aphids (Ali & Agrawal, 2014). Likewise, in potato plants (*Solanum tuberosum*) feeding by *M. persicae* induced solanine levels (potato secondary metabolite) and the induction disappeared upon feeding by the chewer *Leptinotarsa decemlineata* (Davidson‐Lowe *et al*., 2019). The effect of mixed feeding guild attacks may be different across plant species. For example, tomato plants simultaneously attacked by aphids and chewers showed a similar defence response compared to plants only damaged by chewers (Rodriguez‐Saona *et al*., 2005).

Our study revealed that species richness and trait composition of the attackers as a component of multi‐herbivore attack may affect plant induced responses and resistance to subsequent herbivores. We show canalization of plant defences in which plant responses to an increasing species richness in phloem feeders induced vulnerability to larvae of *P. xylostella*. The canalization response suggests that initial attack determines the plant phenotype and reduces potential to convert an induced phenotype in a direction that maximizes resistance to subsequent attack (Agrawal, 2001; Viswanathan *et al*., 2007; Poelman *et al*., 2008; Utsumi *et al*., 2010; Stam *et al*., 2014). However, in cases of facing simultaneous attack by herbivores from different feeding guilds, *B. nigra* plants integrate responses to both feeding guilds, showing an intermediate phenotype. In our study, integration of plant responses to phloem feeders and leaf chewers cancels out the effect of chewer‐induced resistance to larvae of *P. xylostella* to levels of resistance of plants that were not previously attacked by herbivores. Although our current study only scratches the surface of plant resistance strategies to multi‐herbivore attack by revealing a role for species richness, it should be a starting point to further explore the importance of herbivore traits in multi‐herbivore attack such as their level of food plant specialization, order and timing of arrival. Disentangling the role of individual species in the context of natural communities from community processes driven by biodiversity components of multi‐species interactions *per se* may provide deeper understanding of how plant defences evolve under community complexity. Critical steps that should be taken are to evaluate whether plant plasticity is adaptive to specific and perhaps predictable sequences of attack or whether plants can rely on less specified plastic responses to retain fitness. This not only requires to move to challenging plants with complex orders of attackers in controlled glasshouse studies, but to match these patterns with their natural context and assess fitness outcomes in these interactions. Plants may be selected for induced responses that match the patterns of multi‐herbivore attack in which the response to one herbivore should not compromise resistance to likely future attack (Mertens *et al*., 2021). We need to identify the repertoire of defence strategies that plants may use in different ecological settings to understand evolution of plastic defence strategies. Our study also identifies how challenging it is to compromise experimental design to unravel these plant strategies, which should nonetheless be taken to make it feasible to explore the scope of plant plasticity to multi‐herbivore attack.

## Author contributions

MFB, MD and EHP planned and designed the study. MFB collected most data. MEB collected data on insect performance for approach 1 for the performance of *P. xylostella* on plants induced by leaf‐chewer and mixed herbivores and together with MFB collected data for the gene expression experiment. SNK collected data on insect performance for approach 1 for the performance of *P. xylostella* on plants induced by phloem feeders. JB designed the primers and assisted during the gene expression experiment. GG carried out the statistical analyses. MFB, MD and EHP wrote the manuscript.

## Supporting information

**Fig. S1** Plant gene expression after 96 h of feeding by a diversity of herbivores of mixed feeding guild.
**Fig. S2** Plant gene expression after 48 h of feeding by leaf chewers.
**Fig. S3** Plant gene expression after 96 h of feeding by leaf chewers.
**Fig. S4** Plant gene expression after 48 h of feeding by phloem feeders.
**Fig. S5** Plant gene expression after 96 h of feeding by phloem feeders.
**Table S1** Overview of insect combinations for the performance mix species richness experiment for approach 1 showing the number of plant replicates per species richness.
**Table S2** Overview of plant replicates per treatment and per species richness within each feeding guild for approach 2: direct comparison of *Plutella xylostella* performance on phloem feeder, caterpillar and mixed herbivore induced *Brassica nigra* plants.
**Table S3** Primer sequences for the molecular analysis of *Brassica nigra* genes of interest and reference genes.
**Table S4** Statistical output of the models for testing for differences on performance of *Plutella xylostella*.
**Table S5** Significance differences of *Plutella xylostella* weight on plants previously attacked by a mix of leaf chewers and phloem feeders for approach 1.
Please note: Wiley Blackwell are not responsible for the content or functionality of any Supporting Information supplied by the authors. Any queries (other than missing material) should be directed to the *New Phytologist* Central Office.

## References

[nph17228-bib-0001] Acevedo FE, Rivera‐Vega LJ, Chung SH, Ray S, Felton GW. 2015. Cues from chewing insects – the intersection of DAMPs, HAMPs, MAMPs and effectors. Current Opinion in Plant Biology 26: 80–86. 26123394 10.1016/j.pbi.2015.05.029

[nph17228-bib-0002] Agrawal AA. 1999. Induced responses to herbivory in wild radish: effects on several herbivores and plant fitness. Ecology 80: 1713–1723.

[nph17228-bib-0003] Agrawal AA. 2000. Specificity of induced resistance in wild radish: causes and consequences for two specialist and two generalist caterpillars. Oikos 89: 493–500.

[nph17228-bib-0004] Agrawal AA. 2001. Phenotypic plasticity in the interactions and evolution of species. Science 294: 321–326. 11598291 10.1126/science.1060701

[nph17228-bib-0005] Ahman I, Kim SY, Zhu LH. 2019. Plant genes benefitting aphids‐potential for exploitation in resistance breeding. Frontiers in Plant Science 10: 14. 31798609 10.3389/fpls.2019.01452 PMC6874142

[nph17228-bib-0006] Ali JG, Agrawal AA. 2014. Asymmetry of plant‐mediated interactions between specialist aphids and caterpillars on two milkweeds. Functional Ecology 28: 1404–1412.

[nph17228-bib-0007] Arena GD, Ramos‐Gonzalez PL, Rogerio LA, Ribeiro‐Alves M, Casteel CL, Freitas‐Astua J, Machado MA. 2018. Making a better home: modulation of plant defensive response by *Brevipalpus* mites. Frontiers in Plant Science 9: 19. 30158942 10.3389/fpls.2018.01147 PMC6104575

[nph17228-bib-0008] Bell E, Creelman RA, Mullet JE. 1995. A chloroplast lipoxygenase is required for wound‐induced jasmonic acid accumulation in Arabidopsis. Proceedings of the National Academy of Sciences, USA 92: 8675–8679. 10.1073/pnas.92.19.8675 PMC41029 7567995

[nph17228-bib-0009] Berg G. 2009. Plant‐microbe interactions promoting plant growth and health: perspectives for controlled use of microorganisms in agriculture. Applied Microbiology and Biotechnology 84: 11–18. 19568745 10.1007/s00253-009-2092-7

[nph17228-bib-0011] Davidson‐Lowe E, Szendrei Z, Ali JG. 2019. Asymmetric effects of a leaf‐chewing herbivore on aphid population growth. Ecological Entomology 44: 81–92.

[nph17228-bib-0012] Elzinga DA, De Vos M, Jander G. 2014. Suppression of plant defenses by a *Myzus persicae* (Green Peach Aphid) salivary effector protein. Molecular Plant–Microbe Interactions 27: 747–756. 24654979 10.1094/MPMI-01-14-0018-R PMC4170801

[nph17228-bib-0013] Elzinga DA, Jander G. 2013. The role of protein effectors in plant–aphid interactions. Current Opinion in Plant Biology 16: 451–456. 23850072 10.1016/j.pbi.2013.06.018

[nph17228-bib-0015] Erb M, Reymond P. 2019. Molecular interactions between plants and insect herbivores. Annual Review of Plant Biology 70: 527–557. 10.1146/annurev-arplant-050718-095910 30786233

[nph17228-bib-0016] Giordanengo P, Brunissen L, Rusterucci C, Vincent C, van Bel A, Dinant S, Girousse C, Faucher M, Bonnemain JL. 2010. Compatible plant–aphid interactions: how aphids manipulate plant responses. Comptes Rendus Biologies 333: 516–523. 20541163 10.1016/j.crvi.2010.03.007

[nph17228-bib-0017] Giron D, Dubreuil G, Bennett A, Dedeine F, Dicke M, Dyer LA, Erb M, Harris MO, Huguet E, Kaloshian I*et al*. 2018. Promises and challenges in insect–plant interactions. Entomologia Experimentalis Et Applicata 166: 319–343.

[nph17228-bib-0018] Heidel AJ, Baldwin IT. 2004. Microarray analysis of salicylic acid‐ and jasmonic acid‐signalling in responses of *Nicotiana attenuata* to attack by insects from multiple feeding guilds. Plant, Cell & Environment 27: 1362–1373.

[nph17228-bib-0020] Jiang Y, Zhange C‐X, Chenf R, He SY. 2019. Challenging battles of plants with phloem‐feeding insects and prokaryotic pathogens. Proceedings of the National Academy of Sciences, USA 116: 23390–23397. 10.1073/pnas.1915396116 PMC6876188 31712429

[nph17228-bib-0021] Karban R. 2019. The ecology and evolution of induced responses to herbivory and how plants perceive risk. Ecological Entomology 45: 1–9.

[nph17228-bib-0022] Kielkiewicz M, Barczak‐Brzyzek A, Karpinska B, Filipecki M. 2019. Unravelling the complexity of plant defense induced by a simultaneous and sequential mite and aphid Infestation. International Journal of Molecular Sciences 20: 12. 10.3390/ijms20040806 PMC6412847 30781828

[nph17228-bib-0023] Koornneef A, Pieterse CMJ. 2008. Cross talk in defense signaling. Plant Physiology 146: 839–844. 18316638 10.1104/pp.107.112029 PMC2259093

[nph17228-bib-0025] Kroes A, van Loon JJA, Dicke M. 2015. Density‐dependent interference of aphids with caterpillar‐induced defenses in Arabidopsis: involvement of phytohormones and transcription factors. Plant and Cell Physiology 56: 98–106. 25339349 10.1093/pcp/pcu150

[nph17228-bib-0026] Lankau RA, Strauss SY. 2008. Community complexity drives patterns of natural selection on a chemical defense of *Brassica nigra* . American Naturalist 171: 150–161. 10.1086/524959 18197768

[nph17228-bib-0027] Li B, Förster C, Robert CAM, Züst T, Hu L, Machado RAR, Berset JD, Handrick V, Knauer T, Hensel G*et al*. 2018. Convergent evolution of a metabolic switch between aphid and caterpillar resistance in cereals. Science Advances 4: eaat6797. 30525102 10.1126/sciadv.aat6797 PMC6281429

[nph17228-bib-0028] Li J, Brader G, Palva ET. 2004. The WRKY70 transcription factor: a node of convergence for jasmonate‐mediated and salicylate‐mediated signals in plant defense. The Plant Cell 16: 319–331. 14742872 10.1105/tpc.016980 PMC341906

[nph17228-bib-0030] Li YH, Dicke M, Kroes A, Liu W, Gols R. 2016. Interactive effects of cabbage aphid and caterpillar herbivory on transcription of plant genes associated with phytohormonal signalling in wild cabbage. Journal of Chemical Ecology 42: 793–805. 27530535 10.1007/s10886-016-0738-3 PMC5045842

[nph17228-bib-0031] Li YH, Stam JM, Poelman EH, Dicke M, Gols R. 2016. Community structure and abundance of insects in response to early‐season aphid infestation in wild cabbage populations. Ecological Entomology 41: 378–388.

[nph17228-bib-0032] Loreau A, Hector A. 2011. Partitioning selection and complementarity in biodiversity experiments. Nature 412: 72–76. 10.1038/35083573 11452308

[nph17228-bib-0033] Mathur V, Tytgat TOG, de Graaf RM, Kalia V, Reddy AS, Vet LEM, van Dam NM. 2013. Dealing with double trouble: consequences of single and double herbivory in *Brassica juncea* . Chemoecology 23: 71–82.

[nph17228-bib-0034] Mertens D, Boege K, Kessler A, Koricheva J, Thaler JS, Whiteman NK, Poelman EH. 2021. Predictability of biotic stress structures plant defence evolution. Trends in Ecology and Evolution 36: 444–456. 33468354 10.1016/j.tree.2020.12.009 PMC8046718

[nph17228-bib-0035] Moreira X, Abdala‐Roberts L, Hernandez‐Cumplido J, Cuny MAC, Glauser G, Benrey B. 2015. Specificity of induced defenses, growth, and reproduction in lima bean (*Phaseolus lunatus*) in response to multispecies herbivory. American Journal of Botany 102: 1300–1308. 26290553 10.3732/ajb.1500255

[nph17228-bib-0036] Moreira X, Abdala‐Roberts L, Castagneyrol B. 2018. Interactions between plant defence signalling pathways: evidence from bioassays with insect herbivores and plant pathogens. Journal of Ecology 106: 1–12.

[nph17228-bib-0037] Mutti NS, Louis J, Pappan LK, Pappan K, Begum K, Chen MS, Park Y, Dittmer N, Marshall J, Reese JC*et al*. 2008. A protein from the salivary glands of the pea aphid, *Acyrthosiphon pisum*, is essential in feeding on a host plant. Proceedings of the National Academy of Sciences, USA 105: 9965–9969. 10.1073/pnas.0708958105 PMC2481341 18621720

[nph17228-bib-0038] Onkokesung N, Baldwin IT, Gális I. 2010. The role of jasmonic acid and ethylene crosstalk in direct defense of *Nicotiana attenuata* plants against chewing herbivores. Plant Signaling & Behavior 5: 1305–1307. 20930539 10.4161/psb.5.10.13124 PMC3115374

[nph17228-bib-0039] Onkokesung N, Reichelt M, van Doorn A, Schuurink RC, Dicke M. 2016. Differential costs of two distinct resistance mechanisms induced by different herbivore species in Arabidopsis. Plant Physiology 170: 891–906. 26603653 10.1104/pp.15.01780 PMC4734589

[nph17228-bib-0041] Pieterse CMJ, Van der Does D, Zamioudis C, Leon‐Reyes A, Van Wees SCM. 2012. Hormonal modulation of plant immunity. Annual Review of Cell and Developmental Biology 28: 489–521. 10.1146/annurev-cellbio-092910-154055 22559264

[nph17228-bib-0042] Pineda A, Zheng SJ, van Loon JJ, Pieterse CM, Dicke M. 2010. Helping plants to deal with insects: the role of beneficial soil‐borne microbes. Trends in Plant Science 15: 507–514. 20542720 10.1016/j.tplants.2010.05.007

[nph17228-bib-0043] Poelman EH, Kessler A. 2016. Keystone herbivores and the evolution of plant defenses. Trends in Plant Science 21: 477–485. 26832946 10.1016/j.tplants.2016.01.007

[nph17228-bib-0044] Poelman EH, van Loon JJ, Dicke M. 2008. Consequences of variation in plant defense for biodiversity at higher trophic levels. Trends in Plant Science 13: 534–541. 18774329 10.1016/j.tplants.2008.08.003

[nph17228-bib-0045] Poelman EH, Oduor AMO, Broekgaarden C, Hordijk CA, Jansen JJ, Van Loon JJA, Van Dam NM, Vet LEM, Dicke M. 2009. Field parasitism rates of caterpillars on *Brassica oleracea* plants are reliably predicted by differential attraction of *Cotesia* parasitoids. Functional Ecology 23: 951–962.

[nph17228-bib-0046] R Core Team . 2019. R: a language and environment for statistical computing, R v.3.6.1. Vienna, Austria: R Foundation for Statistical Computing.

[nph17228-bib-0047] Rasmann S, Chassin E, Bilat J, Glauser G, Reymond P. 2015. Trade‐off between constitutive and inducible resistance against herbivores is only partially explained by gene expression and glucosinolate production. Journal of Experimental Botany 66: 2527–2534. 25716695 10.1093/jxb/erv033 PMC4986863

[nph17228-bib-0048] Rodriguez‐Saona C, Chalmers JA, Raj S, Thaler JS. 2005. Induced plant responses to multiple damagers: differential effects on an herbivore and its parasitoid. Oecologia 143: 566–577. 15791425 10.1007/s00442-005-0006-7

[nph17228-bib-0049] Rodriguez‐Saona C, Crafts‐Brandner SJ, Canas LA. 2003. Volatile emissions triggered by multiple herbivore damage: beet armyworm and whitefly feeding on cotton plants. Journal of Chemical Ecology 29: 2539–2550. 14682532 10.1023/a:1026314102866

[nph17228-bib-0050] Rodriguez‐Saona CR, Musser RO, Vogel H, Hum‐Musser SM, Thaler JS. 2010. Molecular, biochemical, and organismal analyses of tomato plants simultaneously attacked by herbivores from two feeding guilds. Journal of Chemical Ecology 36: 1043–1057. 20820890 10.1007/s10886-010-9854-7

[nph17228-bib-0051] Soler R, Badenes‐Pérez FR, Broekgaarden C, Zheng S‐J, David A, Boland W, Dicke M. 2012. Plant‐mediated facilitation between a leaf‐feeding and a phloem‐feeding insect in a brassicaceous plant: from insect performance to gene transcription. Functional Ecology 26: 156–166.

[nph17228-bib-0052] Soliveres S, van der Plas F, Manning P, Prati D, Gossner MM, Renner SC, Alt F, Arndt H, Baumgartner V, Bikenstein J*et al*. 2016. Biodiversity at multiple trophic levels is needed for ecosystem multifunctionality. Nature 536: 456–459. 27533038 10.1038/nature19092

[nph17228-bib-0053] Stam JM, Chretien L, Dicke M, Poelman EH. 2017. Response of *Brassica oleracea* to temporal variation in attack by two herbivores affects preference and performance of a third herbivore. Ecological Entomology 42: 803–815. 29200601 10.1111/een.12455 PMC5698737

[nph17228-bib-0054] Stam JM, Dicke M, Poelman EH. 2018. Order of herbivore arrival on wild cabbage populations influences subsequent arthropod community development. Oikos 127: 1482–1493.

[nph17228-bib-0055] Stam JM, Kos M, Dicke M, Poelman EH. 2019. Cross‐seasonal legacy effects of arthropod community on plant fitness in perennial plants. Journal of Ecology 107: 2451–2463. 10.1111/1365-2745.13231 PMC6774310 31598003

[nph17228-bib-0056] Stam JM, Kroes A, Li Y, Gols R, van Loon JJ, Poelman EH, Dicke M. 2014. Plant interactions with multiple insect herbivores: from community to genes. Annual Review of Plant Biology 65: 689–713. 10.1146/annurev-arplant-050213-035937 24313843

[nph17228-bib-0057] Thaler JS, Humphrey PT, Whiteman NK. 2012. Evolution of jasmonate and salicylate signal crosstalk. Trends in Plant Science 17: 260–270. 22498450 10.1016/j.tplants.2012.02.010

[nph17228-bib-0058] Utsumi S, Ando Y, Miki T. 2010. Linkages among trait‐mediated indirect effects: a new framework for the indirect interaction web. Population Ecology 52: 485–497.

[nph17228-bib-0059] Viswanathan DV, Lifchits OA, Thaler JS. 2007. Consequences of sequential attack for resistance to herbivores when plants have specific induced responses. Oikos 116: 1389–1399.

[nph17228-bib-0060] Walling LL. 2000. The myriad plant responses to herbivores. Journal of Plant Growth Regulation 19: 195–216. 11038228 10.1007/s003440000026

[nph17228-bib-0061] Wasternack C, Song SS. 2017. Jasmonates: biosynthesis, metabolism, and signaling by proteins activating and repressing transcription. Journal of Experimental Botany 68: 1303–1321. 27940470 10.1093/jxb/erw443

[nph17228-bib-0062] Will T, Tjallingii WF, Thonnessen A, van Bel AJE. 2007. Molecular sabotage of plant defense by aphid saliva. Proceedings of the National Academy of Sciences, USA 104: 10536–10541. 10.1073/pnas.0703535104 PMC1965548 17553961

[nph17228-bib-0063] Williams L, Rodriguez‐Saona C, Pare PW, Crafts‐Brandner SJ. 2005. The piercing‐sucking herbivores *Lygus hesperus* and *Nezara viridula* induce volatile emissions in plants. Archives of Insect Biochemistry and Physiology 58: 84–96. 15660365 10.1002/arch.20035

[nph17228-bib-0064] Wise MJ, Rausher MD. 2013. Evolution of resistance to a multiple‐herbivore community: genetic correlations, diffuse coevolution, and constraints on the plants response to selection. Evolution 67: 1767–1779. 23730768 10.1111/evo.12061

[nph17228-bib-0065] Zarate SI, Kempema LA, Walling LL. 2007. Silverleaf whitefly induces salicylic acid defenses and suppresses effectual jasmonic acid defenses. Plant Physiology 143: 866–875. 17189328 10.1104/pp.106.090035 PMC1803729

[nph17228-bib-0067] Züst T, Agrawal AA. 2016. Mechanisms and evolution of plant resistance to aphids. Nature Plants 2: 15206. 27250753 10.1038/nplants.2015.206

