# Supplementary material for: Insect species richness affects plant responses to multi‐herbivore attack
Source: New Phytol. 2021 Feb 15;231(6):2333–45. doi: 10.1111/nph.17228 (PMC8451852; doi:10.1111/nph.17228)
Supplement: Supplementary file 1 — Fig. S1 Plant gene expression after 96 h of feeding by a diversity of herbivores of mixed feeding guild. Fig. S2 Plant gene expression after 48 h of feeding by leaf chewers. Fig. S3 Plant gene expression after 96 h of feeding by leaf chewers. Fig. S4 Plant gene expression after 48 h of feeding by phloem feeders. Fig. S5 Plant gene expression after 96 h of feeding by phloem feeders. Table S1 Overview of insect combinations for the performance mix species richness experiment for approach 1 showing the number of plant replicates per species richness. Table S2 Overview of plant replicates per treatment and per species richness within each feeding guild for approach 2: direct comparison of Plutella xylostella performance on phloem feeder, caterpillar and mixed herbivore induced Brassica nigra plants. Table S3 Primer sequences for the molecular analysis of Brassica nigra genes of interest and reference genes. Table S4 Statistical output of the models for testing for differences on performance of Plutella xylostella. Table S5 Significance differences of Plutella xylostella weight on plants previously attacked by a mix of leaf chewers and phloem feeders for approach 1. Please note: Wiley Blackwell are not responsible for the content or functionality of any Supporting Information supplied by the authors. Any queries (other than missing material) should be directed to the New Phytologist Central Office. [file NPH-231-2333-s001.pdf]

New Phytologist **Supporting Information**

Article title: **Insect species richness affects plant responses to multi-herbivore attack**

Authors: Fernández de Bobadilla, Maite; Bourne, Mitchel E.; Bloem, Janneke; Kalisvaart, Sarah N., Gort, Gerrit; Dicke, Marcel; Poelman, Erik H.

Article acceptance date: 14 January 2021

The following Supporting Information is available for this article:

<u>Short legends:</u>

Figure S1. Relative gene expression of *Brassica nigra* leaves at 96h after infesting them with an increasing species richness (1, 2 or 4 species) of aphids, chewers, a mix of both or untreated plants (Ctrl).

Figure S2. Plant gene expression after 48h of feeding by leaf chewers.

Figure S3. Plant gene expression after 96h of feeding by leaf chewers.

Figure S4. Plant gene expression after 48h of feeding by phloem feeders.

Figure S5. Plant gene expression after 96h of feeding by phloem feeders.

Table S1. Overview of insect combinations for the performance mix species richness experiment for approach 1, showing the number of plant replicates per species richness.

Table S2. Overview of plant replicates per treatment and per species richness within each feeding guild for the approach 2: direct comparison of *Plutella xylostella* performance on phloem feeder, caterpillar and mixed herbivore induced *Brassica nigra* plants.

Table S3. Primer sequences for the molecular analysis of *Brassica nigra* genes of interest and reference genes.

Table S4. Variance components of the random factors of the statistical models used for testing for differences on performance of *P. xylostella*

Table S5. Significance differences of *Plutella xylostella* weight on plants previously attacked by a mix of leaf chewers and phloem feeders for approach 1.

<u>Full legends:</u>

Fig. S1 Relative gene expression of *Brassica nigra* leaves at 96h after infesting them with an increasing species richness (1, 2 or 4 species) of aphids, chewers, a mix of both or untreated plants (Ctrl). We measured the expression of JA-biosynthesis and JA-responsive genes *LOX2*, and *VSP2* **(a)** and **(b)**, and the SA-biosynthesis and SA-responsive genes *ICS* and *PR1* **(c)** and **(d)**. Bars represent mean ± SE of log transformed data. Gene expression is relative to the expression level of two reference genes *GAPDH* and *SAR1A*. Bars not sharing letters are significantly different from each other (LM, post hoc LSD).

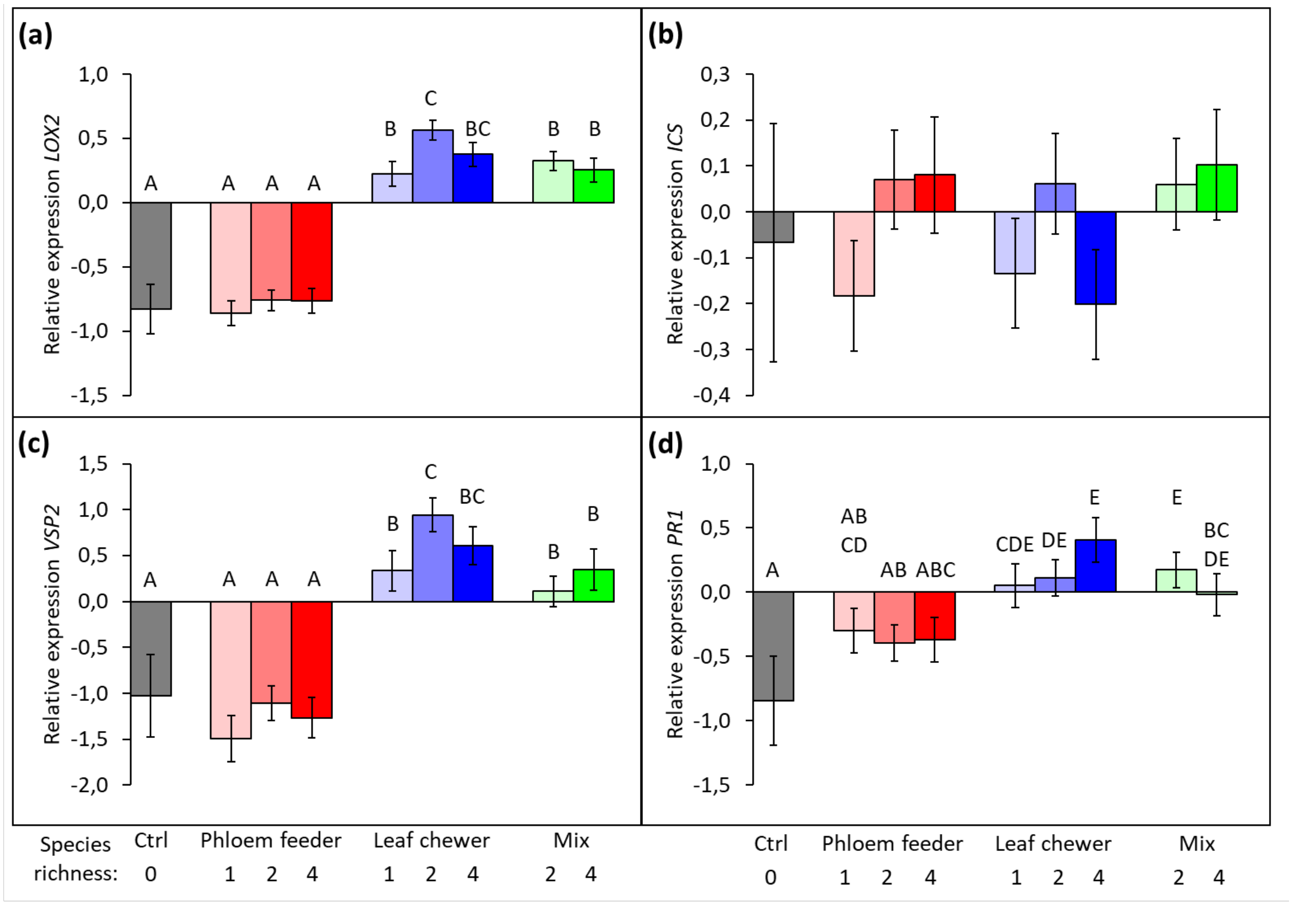

Fig. S2 Relative gene expression of *Brassica nigra* leaves at 48h after infesting them with an increasing species richness (1, 2 or 4 species) of the leaf chewers *Mamestra brassicae* (*Mb*), *Phaedon cochleariae* (*Pc*), *Pieris brassicae* (*Pb*), *Athalia rosae* (*Ar*) alone (Chew.1), in pairs of 2 species (Chew.2), the four species (Chew.4) or untreated plants (Ctrl). We measured the expression of JA biosynthesis and JA responsive genes *LOX2*, and *VSP2* **(a)** and **(b)**, and the SA-biosynthesis and SA-responsive genes *ICS* and *PR1* **(c)** and **(d)**. Bars represent mean ± SE of log transformed data. Gene expression is relative to the expression level of two reference genes *GAPDH* and *SAR1A*. Bars not sharing letters are significantly different from each other (LM, post hoc LSD). Upper case letters above the bars represent differences between treatments. "n.s" not significant. Lower case letters bellow bar-groups represent differences between species richness levels.

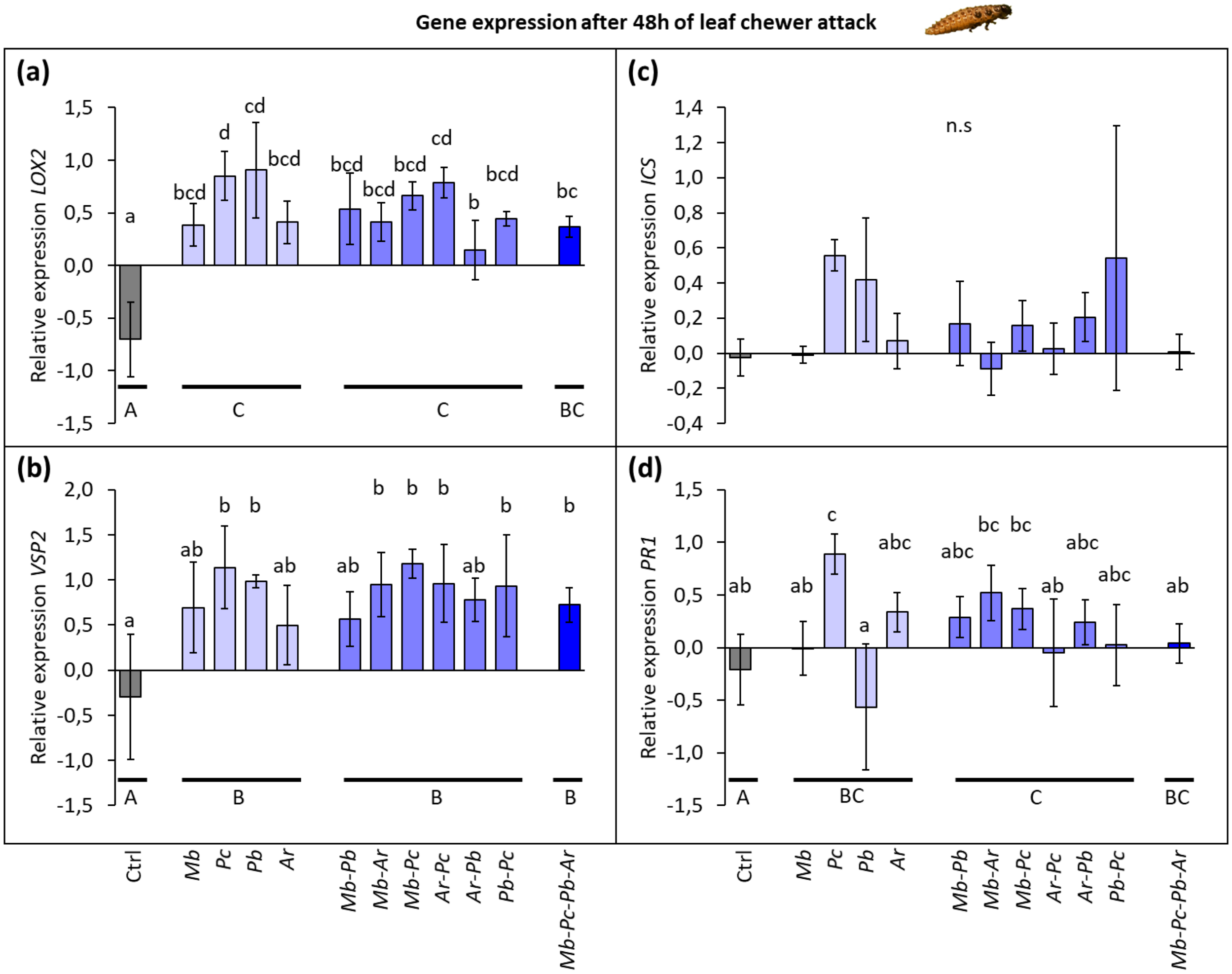

Fig. S3 Relative gene expression of *Brassica nigra* leaves at 96h after infesting them with an increasing species richness (1, 2 or 4 species) of the leaf chewers *Mamestra brassicae* (*Mb*), *Phaedon cochleariae* (*Pc*), *Pieris brassicae* (*Pb*), *Athalia rosae* (*Ar*) alone (Chew.1), in pairs of 2 species (Chew.2), the four species (Chew.4) or untreated plants (Ctrl). We measured the expression of JA-biosynthesis and JA-responsive genes *LOX2*, and *VSP2* **(a)** and **(b)**, and the SA-biosynthesis and SA-responsive genes *ICS* and *PR1* **(c)** and **(d)**. Bars represent mean ± SE of log transformed data. Gene expression is relative to the expression level of two reference genes *GAPDH* and *SAR1A*. Bars not sharing letters are significantly different from each other (LM, post hoc LSD). “n.s” not significant. Upper case letters above the bars represent differences between treatments. Lower case letters bellow bar-groups represent differences between species richness levels.

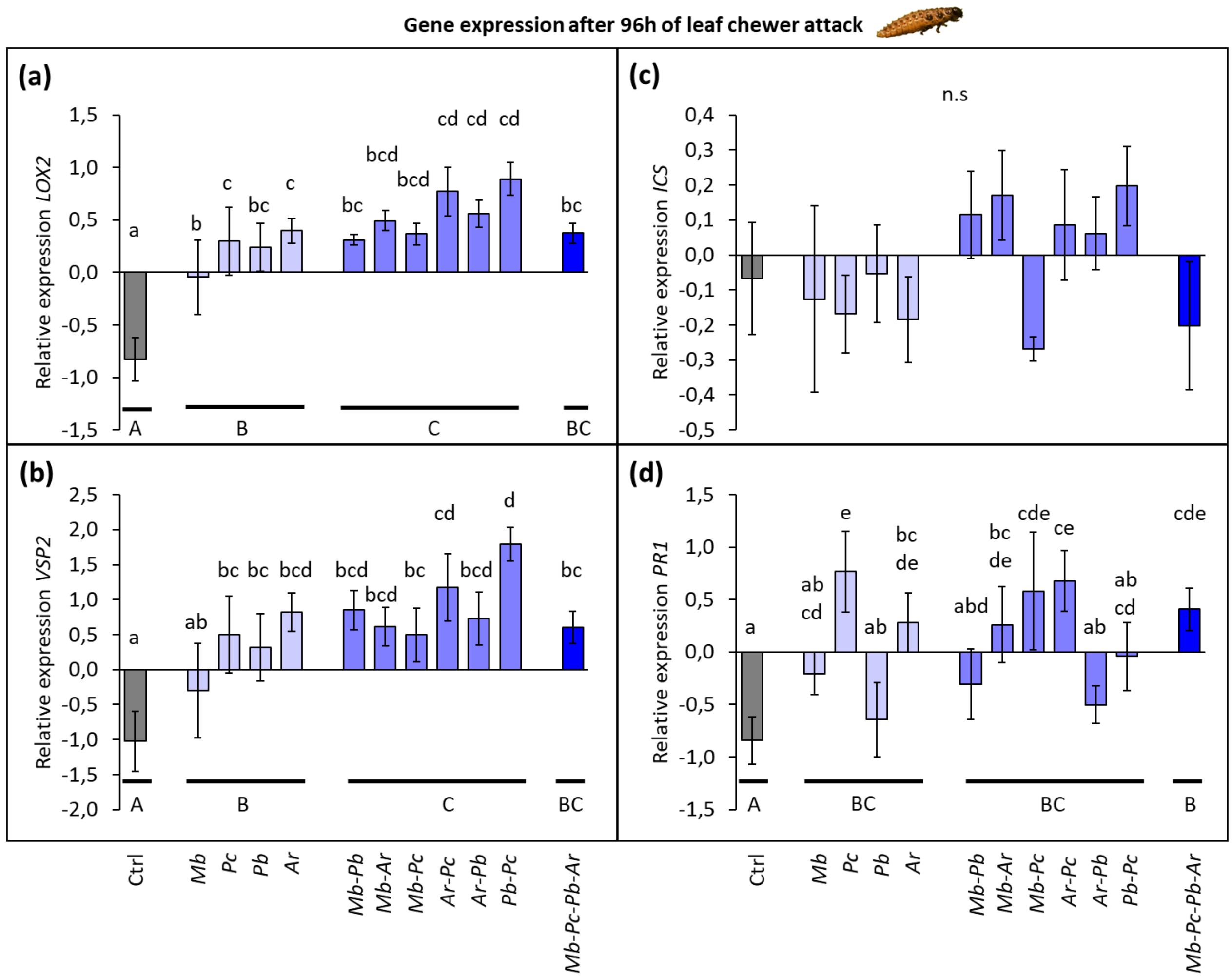

Fig. S4 Relative gene expression of *Brassica nigra* leaves at 48h after infesting them with an increasing species richness (1, 2 or 4 species) of the phloem-feeding aphids *Myzus persicae* sub. *nicotianae* (*Mpn*), *Lipaphis erysimi* (*Le*), *Myzus persicae* (*Mp*), *Brevicoryne brassicae* (*Bb*), alone (Phlo.1), in pairs of 2 species (Phlo.2), the four species (Phlo.4) or untreated plants (Ctrl). We measured the expression of JA-biosynthesis and JA-responsive genes *LOX2*, and *VSP2* **(a)** and **(b)**, and the SA-biosynthesis and SA-responsive genes *ICS* and *PR1* **(c)** and **(d)**. Bars represent mean ± SE of log transformed data. Gene expression is relative to the expression level of two reference genes *GAPDH* and *SAR1A*. Bars not sharing letters are significantly different from each other (LM, post hoc LSD). "n.s" not significant. Upper case letters above the bars represent differences between treatments. Lower case letters bellow bar-groups represent differences between species richness levels.

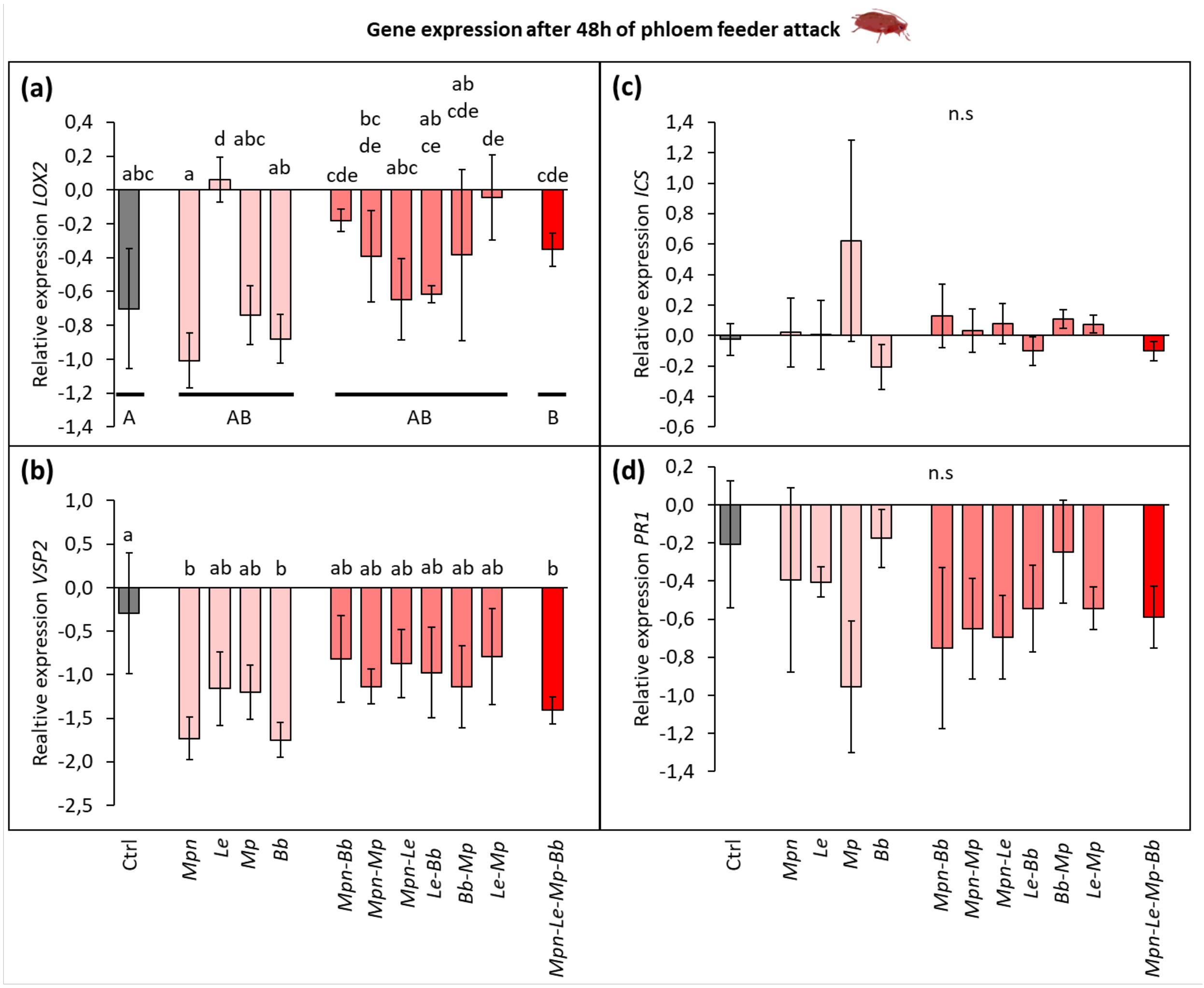

Fig. S5 Relative gene expression of *Brassica nigra* leaves at 96h after infesting them with an increasing species richness (1, 2 or 4 species) of the phloem feeding aphids *Myzus persicae* sub. *nicotianae* (*Mpn*), *Lipaphis erysimi* (*Le*), *Myzus persicae* (*Mp*), *Brevicoryne brassicae* (*Bb*), alone (Phlo.1), in pairs of 2 species (Phlo.2), the four species (Phlo.4) or untreated plants (Ctrl). We measured the expression of JA-biosynthesis and JA-responsive genes *LOX2*, and *VSP2* **(a)** and **(b)**, and the SA-biosynthesis and SA-responsive genes *ICS* and *PR1* **(c)** and **(d)**. Bars represent mean ± SE of log transformed data. Gene expression is relative to the expression level of two reference genes *GAPDH* and *SAR1A*. Bars not sharing letters are significantly different from each other (LM, post hoc LSD). "n.s" not significant. Upper case letters above the bars represent differences between treatments. Lower case letters bellow bar-groups represent differences between species richness levels.

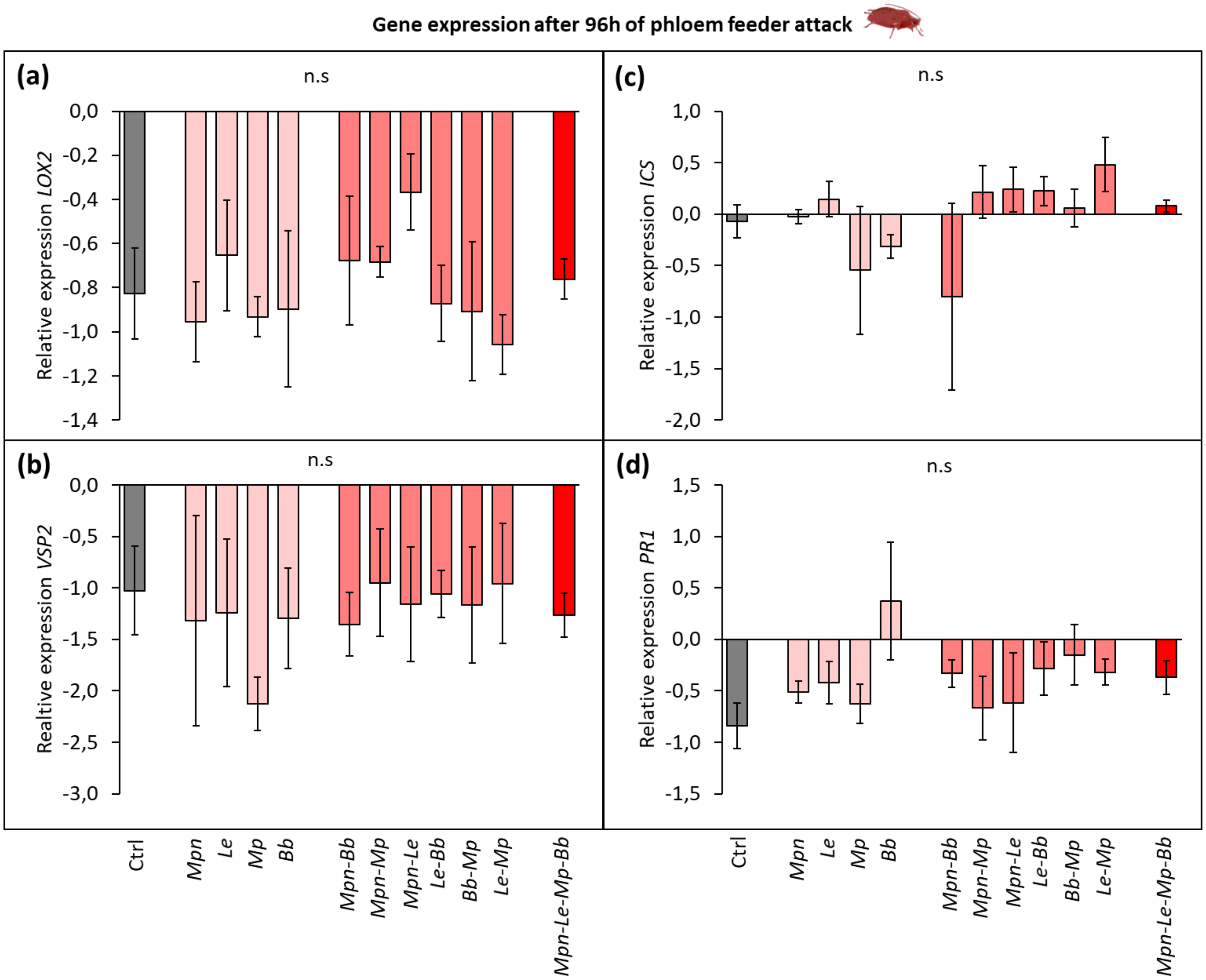

Table S1 Overview of insect combinations for the performance mix species richness experiment for approach 1. showing the number of plant replicates per species richness. We prepared a total of eight plants for each individual insect combination. For Mix.2 we did all the possible insect combinations. For Mix.4 we selected 12 out of the 36 possible combinations (underlined and bold). We did two consecutive sub experiments, preparing half of the replicates for each sub experiment. *Bb= Brevicoryne brassicae, Mp= Myzus persicae, Mpn= Myzus persicae* sub. *nicotianae, Le= Lipaphis erysismi, Ar= Athalia rosae, Pb= Pieris brassicae, Pc= Phaedon cochleariae, Mb=Mamestra brassicae.*

**Con.0**<br>1 treatment<br>10 plants	**Mix.2**<br>16 treatments<br>128 plants		**Mix.4**<br>12 treatments<br>96 plants	
			Bb-Mp-Mb-Ar	*Mp-Mpn-Mb-Ar*
	Bb-Mb	*Mpn-Mb*	*Bb-Mp-Mb-Pb*	***Mp-Mpn-Mb-Pb***
	Bb-Ar	*Mpn-Ar*	*Bb-Mp-Mb-Pc*	*Mp-Mpn-Mb-Pc*
Ctrl	*Bb-Pb*	*Mpn-Pb*	*Bb-Mp-Ar-Pb*	*Mp-Mpn-Ar-Pb*
	Bb-Pc	*Mpn-Pc*	*Bb-Mp-Ar-Pc*	***Mp-Mpn-Ar-Pc***
			Bb-Mp-Pb-Pc	*Mp-Mpn-Pb-Pc*
			Bb-Mpn-Mb-Ar	*Mp-Le-Mb-Ar*
	Mp-Mb	*Le-Mb*	*Bb-Mpn-Mb-Pb*	*Mp-Le-Mb-Pb*
	Mp-Ar	*Le-Ar*	***Bb-Mpn-Mb-Pc***	***Mp-Le-Mb-Pc***
	Mp-Pb	*Le-Pb*	***Bb-Mpn-Ar-Pb***	***Mp-Le-Ar-Pb***
	Mp-Pc	*Le-Pc*	*Bb-Mpn-Ar-Pc*	*Mp-Le-Ar-Pc*
			Bb-Mpn-Pb-Pc	*Mp-Le-Pb-Pc*
			Bb-Le-Mb-Ar	***Mpn-Le-Mb-Ar***
			Bb-Le-Mb-Pb	*Mpn-Le-Mb-Pb*
			Bb-Le-Mb-Pc	*Mpn-Le-Mb-Pc*
			Bb-Le-Ar-Pb	*Mpn-Le-Ar-Pb*
			Bb-Le-Ar-Pc	*Mpn-Le-Ar-Pc*
			Bb-Le-Pb-Pc	***Mpn-Le-Pb-Pc***

Table S2 Overview of plant replicates per treatment and per species richness within each feeding guild for the approach 2: direct comparison of *Plutella xylostella* performance on phloem feeder, chewer and mixed herbivore induced *Brassica nigra* plants. Leaf chewers*: Mb= Mamestra brassicae, Ar= Athalia rosae, Pb= Pieris brassicae, Pc= Phaedon cochleariae*. Phloem feeders*: Bb= Brevicoryne brassicae, Mp= Myzus persicae, Mpn= Myzus persicae* sub. *nicotianae, Le= Lipaphis erysismi.*

Group	Species richness	Treatment	Rep.	Replicates group
Control	0	Con.0	24	24
Chewer	4	Chew. 4	24	24
Phloem feeder	4	Phlo. 4	24	24
Mix	4	*Bb-Mp-Mb-Ar*	2	24
		Bb-Mp-Pb-Pc	2	
		Bb-Mpn-Mb-Pc	2	
		Bb-Mpn-Ar-Pb	2	
		Bb-Le-Mb-Pb	2	
		Bb-Le-Ar-Pc	2	
		Mp-Mpn-Mb-Pb	2	
		Mp-Mpn-Ar-Pc	2	
		Mp-Le-Mb-Pc	2	
		Mp-Le-Ar-Pb	2	
		Mpn-Le-Mb-Ar	2	
		Mpn-Le-Pb-Pc	2	

Table S3 Primer sequences for the molecular analysis of *Brassica nigra* genes of interest and reference genes.

Gene	Pathway	AT	Sequence (Forward, reverse)
LIPOXIGENASE 2<br>*LOX2*	JA biosynthesis	62	TGCTCGTGCACGCCAGAGTC<br>AGCCAGCCCCCTGCTGATGA
VEGETATIVE STORAGE PROTEIN 2<br>*VSP2*	JA responsive	58	TCTACGCCAAAGGACTTG<br>CTCWGTCCCGTATCCATATTGAG
ISOCHORISMATE SYNTHASE<br>*ICS*	SA biosynthesis	58	GCTTGCACAGTTACAGAG<br>CACGCTCTATCTCCATATCAC
PATHOGENESIS-RELATED PROTEIN 1<br>*PR-1*	SA responsive	60	CGCCGACGGACTAAGAGGCG<br>ACACCTCGCTTTGCCACATCCA
WRKY70	SA crosstalk	62	ATGCTTCHTGYGACAACGAC<br>TTTGTTGCCTTGCACCCTTG
SECRETION ASSOCIATED RAS RELATED GTPASE 1A<br>*SAR1A*	Reference	60	ATCTCTAGCCACCGTTCCCT<br>TTCCTGACGATGCTGCACAT
GLYCERALDEHYDE-3-PHOSPHATE DEHYDROGENASE<br>*GAPDH*	Reference	62	GGAGCTGCCAAGGCTGTCGG<br>CCTTCAGATTCCTCCTTGATAGCC
ACTIN-2<br>*ACT-2*	Reference	62	ACATTGTGCTCAGTGGTGGA<br>TCTGCTGGAATGTGCTGAGG
BETA-TUBULINE<br>*B-TUB*	Reference	62	GTCAAGTCCAGCGTCTGTGA<br>TCACACGCCTGAACATCTCC
ELONGATION FACTOR-1<br>*EF-1*	Reference	60	CGTCCCCATCTCTGGATTCG<br>ACAACCATACCGGGCTTGAG
PEROXIDASE 4<br>*PER4*	Reference	62	TATCCTCTGCAGCCTCCTCA<br>ACACACAGACTGAAGCGTCC

Table S4. Variance components of the random factors of the statisticl models used for testing for differences on performance of *P. xylostella*

	With tray			Without tray		
**Variance components**	**s^2**	**Wald Z**	**P**	**s^2**	**Wald Z**	**P**
Block	0.00901	1.14	0.1269	0.00920	1.19	0.1167
Tray	0.00808	3.87	P<0.0001			
Plant	0.00223	2.22	0.0131	0.00715	5.45	P<0.0001
Error(aphids)	0.08366	20.3	P<0.0001	0.08194	20.10	P<0.0001
Error(chewers)	0.05074	22.74	P<0.0001	0.05096	22.51	P<0.0001
Error(mix)	0.09018	23.27	P<0.0001	0.09107	22.94	P<0.0001

Table S5 Significance differences of *Plutella xylostella* weight on plants previously attacked by a mix of leaf chewers and phloem feeders for approach 1. Insect combinations not sharing letters have a significant different effect on *Plutella xylostella* growth (MLM, Tukey post-hoc). Combination of inducers that affected *P. xylostella* growth (compared to control, untreated plants) are marked in bold and with an asterisk. Chewers: *Mamestra brassicae* (*Mb*), *Phaedon cochleariae* (*Pc*), *Pieris brassicae* (*Pb*), *Athalia rosae* (*Ar*). Phloem feeding aphids *Myzus persicae* sub. *nicotianae* (*Mpn*), Lipaphis *erysimi* (*Le*), *Myzus persicae* (*Mp*), *Brevicoryne brassicae* (*Bb*).

Treatment	Significance
Control	bcde
Bb-Ar	ab
Bb-Mb	abcde
Bb-Pb	cdef
Bb-Pc	abcd
Le-Ar	abc
Le-Mb	cdef
Le-Pb	abcd
Le-Pc	abc
Mp-Ar	bcde
Mp-Mb	bcdefg
Mpn-Ar*	**fg**
Mpn-Mb	bcde
Mpn-Pb	bcdefg
Mpn-Pc	cdef
Mp-Pb	cdef
Mp-Pc	bcdefg
Bb-Le-Ar-Pc	cdef
Bb-Le-Pb-Mb	ab
Bb-Mp-Mb-Pc	ef
Bb-Mpn-Ar-Mb	def
Bb-Mpn-Pb-Pc	bcdef

Bb-Mp-Pb-Ar*	**a**
Mp-Le-Ar-Mb	cdef
Mp-Le-Pb-Pc	abcde
Mp-Mpn-Ar-Pc	abc
Mp-Mpn-Pb-Mb	bcdef
Mpn-Le-Mb-Pc*	**f**
Mpn-Le-Pb-Ar	bcdef
